# The *Staphylococcus aureus* ABC-Type Manganese Transporter MntABC Is Critical for Reinitiation of Bacterial Replication Following Exposure to Phagocytic Oxidative Burst

**DOI:** 10.1371/journal.pone.0138350

**Published:** 2015-09-17

**Authors:** Alison Coady, Min Xu, Qui Phung, Tommy K. Cheung, Corey Bakalarski, Mary Kate Alexander, Sophie M. Lehar, Janice Kim, Summer Park, Man-Wah Tan, Mireille Nishiyama

**Affiliations:** 1 Department of Infectious Diseases, Genentech Inc., South San Francisco, California, United States of America; 2 Department of Translational Immunology, Genentech Inc., South San Francisco, California, United States of America; 3 Department of Protein Chemistry, Genentech Inc., South San Francisco, California, United States of America; 4 Department of Bioinformatics, Genentech Inc., South San Francisco, California, United States of America; Institut Pasteur, FRANCE

## Abstract

Manganese plays a central role in cellular detoxification of reactive oxygen species (ROS). Therefore, manganese acquisition is considered to be important for bacterial pathogenesis by counteracting the oxidative burst of phagocytic cells during host infection. However, detailed analysis of the interplay between bacterial manganese acquisition and phagocytic cells and its impact on bacterial pathogenesis has remained elusive for *Staphylococcus aureus*, a major human pathogen. Here, we show that a *mntC* mutant, which lacks the functional manganese transporter MntABC, was more sensitive to killing by human neutrophils but not murine macrophages, unless the *mntC* mutant was pre-exposed to oxidative stress. Notably, the *mntC* mutant formed strikingly small colonies when recovered from both type of phagocytic cells. We show that this phenotype is a direct consequence of the inability of the *mntC* mutant to reinitiate growth after exposure to phagocytic oxidative burst. Transcript and quantitative proteomics analyses revealed that the manganese-dependent ribonucleotide reductase complex NrdEF, which is essential for DNA synthesis and repair, was highly induced in the *mntC* mutant under oxidative stress conditions including after phagocytosis. Since NrdEF proteins are essential for *S*. *aureus* viability we hypothesize that cells lacking MntABC might attempt to compensate for the impaired function of NrdEF by increasing their expression. Our data suggest that besides ROS detoxification, functional manganese acquisition is likely crucial for *S*. *aureus* pathogenesis by repairing oxidative damages, thereby ensuring efficient bacterial growth after phagocytic oxidative burst, which is an attribute critical for disseminating and establishing infection in the host.

## Introduction

*Staphylococcus aureus* is a major cause of nosocomial and community-acquired infections leading high rates of hospitalization and mortality worldwide [1]. A hallmark of *S*. *aureus* as a major human pathogen is its large arsenal of virulence factors involved in colonizing diverse host niches, evading innate immunity, and resisting major antibacterial therapies [2]. Accordingly, *S*. *aureus* is able to infect a wide variety of organs and cause a multitude of diseases such as skin and soft tissue infections, pneumonia, endocarditis and sepsis [3]. As bacteria continue to develop resistance, it is important to define new anti-bacterial targets for the effective treatment of *S*. *aureus* infections. Currently available antibiotics target only a limited number of bacterial cellular functions such as biosynthesis of macromolecules like DNA and protein. Targeting bacterial nutrient uptake systems is an attractive alternative approach, since acquiring essential elements, such as iron and manganese, is critical for the survival and replication of pathogens [4]. In fact, one of the host defense systems to combat infection is to restrict the availability of essential elements from invading pathogens, a process termed “nutritional immunity” [4]. Therefore, a recently identified antibody fragment that interferes with manganese uptake of *S*. *aureus* might provide an alternative therapeutic intervention in treating bacterial infections [5].

While the most prominent example of nutritional immunity is the restriction of iron by the host, recent work has revealed that vertebrates also limit manganese availability during infection [6]. Vertebrates produce two proteins to sequester manganese: calprotectin, a member of the S100 class of EF-hand calcium binding proteins [7], and Nramp1, a multi-spanning integral membrane protein [8]. Both calprotectin and Nramp1 are highly expressed in phagocytic cells that act as a first defense against invading pathogens [6, 9].

The *S*. *aureus* genome encodes for two distinct Mn^2+^ uptake systems: MntABC and MntH [10, 11]. MntABC is an ATP-binding cassette (ABC)-type transporter, which consists of three proteins, the ATP-binding protein MntA, the permease MntB and the metal binding protein MntC, while MntH is a proton-dependent NRAMP transporter. Simultaneous inactivation of MntABC and MntH was shown to attenuate the virulence of the laboratory strain 8325–4 and the methicillin-sensitive clinical strain Newman in a murine skin abscess model and in a murine systemic infection model, respectively [10, 11]. In the methicillin-resistant *S*. *aureus* (MRSA) strain USA300, inactivation of MntABC alone was sufficient to attenuate its virulence in a murine systemic infection model [12]. The differences in the requirement of functional manganese acquisition systems for *S*. *aureus* pathogenesis might be strain- and infection model-dependent, but the functionality of the host *Nramp1* locus is known to be important for analyzing manganese-dependent processes of pathogens within the host [13]. While the major laboratory mouse strains such as BALB/c and C57BL/6, which were used in the previous studies [10, 11], do not have a functional *Nramp1* locus, the A/J strain that was used in the latest study does [12, 14]. Functional Mn^2+^ acquisition systems are also critical for full virulence of several other pathogens, including *Neisseria gonorrhoeae* [15], *Salmonella* species [16], and *Streptococcus* species [17], suggesting the universal requirement of manganese for bacterial pathogenesis.

During the oxidative burst (or respiratory burst) of phagocytic cells, a significant amount of reactive oxygen species (ROS) is released [18, 19]. Oxidative burst is initiated by the assembly of the NADPH oxidase complex, which catalyzes the reduction of oxygen (O_2_) to superoxide (O_2_^−^), which is further reduced to hydroxyl radical (OH^·^) or dismutated to hydrogen peroxide (H_2_O_2_) [20]. Moreover, neutrophils (but not macrophages) contain high concentrations of myeloperoxidase (MPO), which together with H_2_O_2_ and Cl^−^ produces the strong oxidant hypochlorous acid (HOCl) [18]. O_2_^−^, OH^·^, H_2_O_2_, and HOCl are ROS and are capable of damaging a number of cellular structures including membranes and macromolecules in particular DNA [21, 22]. Manganese has been proposed to detoxify ROS in various ways: the detoxification of O_2_^−^ by Mn^2+^-dependent superoxide dismutases is the best-characterized Mn^2+^-dependent ROS detoxification mechanism [23]. Indeed, it was shown that concomitant inactivation of both MntC and MntH ablated *S*. *aureus* superoxide dismutase activity under manganese-restricting conditions [11]. In *E*. *coli*, it has been proposed that Mn^2+^ might be able to replace the more reactive Fe^2+^ in Fe^2+^-containing proteins, thereby reducing oxidative damage to these proteins [24]. Non-enzymatic detoxification of ROS by Mn^2+^ has been proposed for *S*. *aureus* and other bacteria [25, 26], but its relevance has been controversially discussed [27]. Since phagocytic cells are the major source of ROS during infection [18], functional manganese acquisition systems are thought to be important for bacterial survival in the host. To date, however, few studies have directly investigated the role functional manganese acquisition plays in the interaction of *S*. *aureus* with phagocytic cells. The majority of published work has focused on *in vitro* experiments where the response of *S*. *aureus* mutant strains towards oxidative stress was investigated using the ROS-generating agent methyl viologen whose mechanism of ROS generation has shown to be distinct from the oxidative burst of phagocytic cells [28–31]. Moreover, the *in vitro* experiments have been usually performed under non-physiological conditions, *e*.*g*. bacteria are incubated with high concentrations of methyl viologen for an extended time, often overnight. Therefore, the goal of our study was to directly investigate the role functional manganese acquisition plays in the interaction of *S*. *aureus* and phagocytic cells. Moreover, we aimed to elucidate the biological processes that are affected by manganese depletion during oxidative stress in order to better understand the molecular basis underlying the strong attenuation of the MRSA strain USA300 lacking MntABC during murine systemic infection [12].

## Materials and Methods

### Ethics Statement

All research involving human participants were approved by Western Institutional Review Board (WIRB). All individuals who donated blood for research use have signed an IRB approved consent form. All procedures involving mice were compliant with the ILAR guidelines, and were approved by the IACUC at Genentech, Inc.

### Bacterial strains

Methicillin-resistant *S*. *aureus* (MRSA) SF8300, a clinical isolate representative of the epidemic strain USA300 [32], and its isogenic *mntC* mutant, in which the *mntC* gene was inactivated by changing its 8th and 12th codon to a stop codon, were obtained as described previously [12].

### Bone marrow derived macrophage isolation

Bone marrow-derived macrophages (BMDMs) were isolated from the femurs of A/J mice purchased from the Jackson Laboratory. Bone marrow was eluted using Bone Marrow Macrophage medium (BMM), consisting of a 50:50 mix of Dulbecco's Modified Eagle Medium and F12 media with 20% heat-inactivated Fetal Bovine Serum (FBS), 100 ng/ml rM-CSF, 2 mM glutamine, 110 μg/ml sodium pyruvate, penicillin and streptomycin. Bone marrow cells were cultured for 6–7 days in a tissue culture incubator. Adherent cells were harvested by gentle scraping after washing with cold PBS (Mg^2+^- and Ca^2+^-free) plus 1 mM EDTA and frozen in FBS + 10% DMSO for storage in liquid nitrogen until use.

### Neutrophil killing assay

Polymorphonuclear leukocytes (PMN) were isolated from heparin-treated whole human blood (from healthy donors) by the addition of an equal volume of 3% Dextran and 0.9% NaCl. After incubation at room temperature for 20 min, PMNs were pelleted from the upper layer by centrifugation at 200 × *g* for 5 min. Cells were washed and resuspended in Hanks Balanced Salt Solution containing 10 mM HEPES pH 7.4 and 1% BSA (HB media). *S*. *aureus* was grown to mid-log phase (OD_600_ = 0.5) in Roswell Park Memorial Institute (RPMI) media (RPMI 1640, Sigma-Aldrich) supplemented with 10 mM HEPES (RPMI-H). *S*. *aureus* cells were spun down and resuspended to an OD_600_ of 0.5 in 10 mL RPMI-H ± 1 μM methyl viologen, then incubated at 37°C with shaking for 1 hour. *S*. *aureus* cells were washed once in fresh RPMI-H, resuspended in 10 ml HB media, and pre-opsonized in 10% human serum at 37°C for 30 min. Bacteria were then added to 9 × 10^4^ PMNs at an MOI of 10 and incubated at 37°C for 90 min in a tissue culture incubator. PMNs were lysed in PBS with 0.1% Triton X-100. Viable bacterial counts were determined by plating serial dilutions on Tryptic Soy Agar (TSA) containing 5% defibrinated sheep’s blood.

### Macrophage killing assay

BMDMs were seeded from frozen stocks into 24-well tissue culture plates at a density of 2 × 10^5^ cells/well in BMM. After 24 hours of culture in a tissue culture incubator, cells were given fresh media plus 10 ng/ml IFNγ (Peprotech) without antibiotics. Macrophages were then washed and infected with prepared *S*. *aureus* cells (±1 μM methyl viologen as described above without pre-opsonization) at an MOI of 10. After incubation at 37°C for 90 min, the inoculum was removed from BMDMs and replaced with BMM containing 50 μg/ml gentamycin to kill extracellular *S*. *aureus* cells. After 24 hours of incubation, bacteria were harvested from macrophages by adding PBS containing 0.1% Triton X-100 to lyse macrophages. Viable bacterial counts were determined by plating serial dilutions on TSA containing 5% defibrinated sheep’s blood.

### CFSE proliferation assay

Bacteria were grown overnight in RPMI medium buffered with 10 mM HEPES pH 7.2, then diluted into fresh media and grown to mid-log phase. Bacteria were then harvested, washed, and resuspended in PBS buffer containing 0.1% Bovine Serum Albumin (PBS-BSA) to a concentration of 1 × 10^9^ cfu/ml. Labeling reaction was carried out at 37°C for 30 minutes with 100 μM carboxyfluorescein diacetate succinimidyl ester (CFSE) (Molecular Probes, Eugene, OR) diluted in PBS-BSA. Excess label was removed by washing 3 times in PBS-BSA. To confirm that the CFSE label was retained in the absence of cell division, bacterial cell division was inhibited by irradiation or co-culturing with high concentrations of unlabeled stationary phase bacteria. For irradiation, bacteria were diluted to 1 × 10^9^ cfu/ml in PBS containing 0.1% BSA. 1 ml of the bacterial suspension was placed in each well of a 24-well tissue culture dish and irradiated 2 times at 400 mJoules using a UV stratalinker 2400 (Stratagene). For co-culturing, 100 μl CFSE-labeled bacteria (1 × 10^8^ cfu/ml) were added directly to a 10 ml overnight culture of unlabeled bacteria (~3 × 10^9^ cfu/ml). Macrophage infection was carried out with CFSE-labeled bacteria as described before with the exception that dH_2_O and agitation were utilized to lyse the macrophages. CFSE-labeled bacteria that were recovered from macrophages were resuspended in TSB media and allowed to grow aerobically at 37°C. Samples were taken at indicated time points and were fixed by the addition of 2% paraformaldehyde in PBS. Retention of CFSE label was determined by flow cytometry using a FACSAria cell sorter (BD Biosciences, San Jose, CA) and data analysis was performed using FlowJo software (Tree Star Inc, Ashland, OR). To distinguish bacteria from macrophage debris during FACS analysis, samples were immunolabeled with an anti-*S*. *aureus* Alexa647-conjugated antibody (Genentech) and gates were set with controls that were singly labeled with either the anti-*S*. *aureus* Alexa647 antibody or with CFSE but not exposed to phagocytosis or conditions that induced proliferation.

### Growth after methyl viologen treatment

*S*. *aureus* was grown to mid-log phase (OD_600_ = 0.5) in RPMI-H. Cells were spun down and resuspended to an OD_600_ of 0.5 in 10 mL RPMI-H ± 1 μM methyl viologen, then incubated at 37°C with shaking for 1 hour. Cells were washed once in fresh RPMI-H, inoculated 1:100 in 10 ml fresh RPMI-H and incubated at 37°C with shaking. At the indicated time points, aliquots of culture were removed and assayed in triplicate for intracellular ATP concentrations using BacTiter-Glo (Promega) according to manufacturer’s instructions with the following modification: samples were incubated in the dark for 15 minutes to ensure lysis of the bacteria. Luminescence was measured on a Synergy 2 Multi-Mode Microplate Reader (BioTek). Bacterial growth was measured as the fold change in luminescence relative to the starting culture.

### Recovery after phagocytosis

To measure the recovery of *S*. *aureus* after phagocytosis, 200 μl aliquots of recovered lysate, which contained comparable amount of wild-type and *mntC* mutant bacteria as determined by Bac-Titer-Glo luminescence, were added to 10 ml of RPMI-H. Bacterial growth was measured at the indicated time points as the fold change in the BacTiter-Glo luminescence relative to the starting culture as described above.

### Harvesting of *S*. *aureus* RNA after methyl viologen treatment

*S*. *aureus* was grown to mid-log phase (OD_600_ = 0.5) in RPMI-H. Cells were spun down and resuspended to an OD_600_ of 0.5 in 10 mL RPMI-H ± 1 μM methyl viologen, then incubated at 37°C with shaking for 1 hour. Cells were washed once in fresh RPMI-H and pelleted by centrifugation at 2400 × *g* for 15 min at 4°C. Bacterial pellets were resuspended in PBS with 2 volumes RNAProtect Bacteria Reagent (Qiagen), and incubated at room temperature with gentle rotation for 5 minutes. Samples were then spun down at 8500 × g for 10 min at 4°C and bacterial pellets were stored at −80°C until RNA isolation. To harvest RNA, bacteria were first incubated with 80 μg lysostaphin (Sigma), 80,000 U RNasin (Promega) and 200 μg Proteinase K (Qiagen) for 30 min. Samples were then lysed via bead-beating using 0.1 mm glass beads (BioSpec) and RNA was subsequently isolated per manufacturer’s instructions using the RNeasy mini kit (Qiagen).

### Harvesting of *S*. *aureus* RNA after phagocytosis

8 × 10^5^ BMDMs or 3.6 × 10^5^ PMNs were seeded in 6 well tissue culture plates and infected with *S*. *aureus* at an MOI of 20 as described above for killing assays. After infection for 45 minutes (PMNs) or 2 hours (macrophages), cells were lysed by incubating with PBS + 0.1% Triton X-100 for 2 min. Lysate from 3 wells was pooled and phagocytosed bacteria were recovered by centrifugation at 2400 × *g* for 20 min at 4°C. Bacterial pellets were resuspended in PBS with 2 volumes RNAProtect Bacteria Reagent (Qiagen) and incubated at room temperature with gentle rotation for 5 min. Samples were then spun down at 8500 × *g* for 10 min at 4°C and bacterial pellets were stored at −80°C until RNA was isolated as described above.

### Analysis of *S*. *aureus* mRNA expression

cDNA was synthesized from 500−1000 ng of Turbo-DNase treated RNA using the Superscript III First-Strand Synthesis Supermix for qRT-PCR (Life Technologies). cDNA reactions were diluted 1:10 using nuclease-free water and analyzed by qRT-PCR. qRT-PCR was performed in triplicate using custom designed Taqman primers on a 7500 Real-Time PCR System (S1 Table) (Applied Biosciences). As an internal control, primers specific for the 16S rRNA were used.

### Cell Culture and SILAC Labeling

Three independent cultures from independent clones of wild-type and *mntC* mutant strains were grown in RPMI 1640 (Thermo Scientific, 89984) media containing 1 μM of methyl viologen supplemented with either light arginine (Sigma, A5006) and lysine (Sigma, L5501) or heavy arginine (Sigma, 608033) and lysine (Sigma, 608041) to OD_600_ of 0.3, washed and resuspended in PBS. Both heavy and light SILAC media were supplemented with light proline (Sigma, P0380). Bacteria were lysed by mechanical disruption using a Mini-Beadbeater (Biospec Products). The lysates were centrifuged at 18,000 × *g* for 10 min. Bradford assays (BioRad) were performed on each lysate to determine the protein concentration.

### SILAC Incorporation Analysis

To determine the incorporation rate of heavy amino acids, 5 μg of lysate of cells grown in heavy media was reduced with 10 mM dithioreitol (DTT) and loaded onto a NuPage 4–12% Bis-Tris gel (Invitrogen). A gel band in the region around 60 kDa from each lane was excised, destained with 50 mM ammonium bicarbonate/50% methanol, and digested with 0.02 μg/μl trypsin (Promega) in 50 mM ammonium bicarbonate overnight at 37°C. Digests were subjected to LC-MS/MS analysis using similar conditions as described earlier [11]. Tandem mass spectral data was submitted for Mascot version 2.3.02 (Matrix Science) database search against a concatenated target-decoy database consisting of *S*. *aureus* proteins from UniProtKB (version 2011_12) and common laboratory contaminants. Database search parameters included full tryptic specificity, variable modifications of heavy lysine (K8, +8.014Da) and heavy arginine (R10, +10.008Da), methionine oxidation (+15.995Da), 2 missed cleavages, and 30 ppm precursor ion mass and 0.5 Da fragment ion mass tolerance. Peptide spectral matches were post-filtered to a 10% peptide false discovery rate using a linear discriminant approach as previously described [33]. Quantification was performed using the VistaQuant [34] algorithm and only high quality peptides with VistaQuant scores of 95 and above were employed to verify SILAC incorporation rates.

### SILAC Proteome Profiling

“Light” wild-type and “Heavy” *mntC* mutant lysates were mixed in a 1:1 ratio based on protein concentration. The reverse labeled lysates “Heavy” wild-type and “Light” *mntC* mutant were also similarly mixed. A total of 60 μg of protein for each mixed SILAC pair was reduced with DTT and loaded onto a 4–12%Bis-Tris gel (Invitrogen). The resulting gel lanes were excised into 15 slices and subjected to in-gel trypsin digestion as described above. Following digestion, each sample was analyzed via LC-MS/MS with duplicate injections using similar conditions as described previously [12], however with a 67 min gradient from 2−90% Buffer B (Buffer B comprises of 2% water/ 98% acetonitrile/ 0.1% formic acid) at 1.00 μl/min with a total analysis time of 90 min. Tandem mass spectral data was submitted for Mascot (Matrix Science) database search against a concatenated target-decoy database consisting of *S*. *aureus* proteins and common laboratory contaminants as described above. The data was searched with full trypsin enzyme specificity, variable modifications of heavy lysine (K8, +8.014Da) and heavy arginine (R10, +10.008Da), and methionine oxidation (+15.995Da), and 30 ppm precursor ion mass and 0.5 Da fragment.

### Protein-level Quantification and Normalization

Quantification was performed using the VistaQuant algorithm [34]. Quantified peptide spectral matches (PSMs) with VistaQuant scores of 83 and above were combined across all gel fractions for a given condition and labeling set and normalized to the median relative abundance ratio of each combined dataset. Log_2_ protein relative abundance ratios were calculated as the log-transformed ratio of the summed area under curve measurements of the labeled and unlabeled peptide species. In instances where multiple quantification events were observed for the same species, as determined by overlapping ion masses and peak retention times, the event with the largest total area under the curve was chosen as representative and all others were discarded. Events for which either the light or heavy species was below the limit of detection of the instrument were set to the signal to noise ratio of the observed species and denoted by an inequality symbol in the results. Standard scores (*z*-scores) for each protein ratio were calculated against a moving standard deviation calculated as a function of overall abundance as previously described [34]. Proteins were stratified into two lists: first, a list for which all absolute protein standard scores when observed were ≥2, and a second slightly relaxed list where at least one observation had an absolute protein standard score ≥2 while requiring that other observations be consistent (that is, the sign of all standard scores for a given protein are the same).

## Results

### Lack of a functional MntABC system renders *S*. *aureus* more sensitive to killing by human neutrophils but not murine macrophages, unless *S*. *aureus* is pre-exposed to oxidative stress

**Fig 1:**
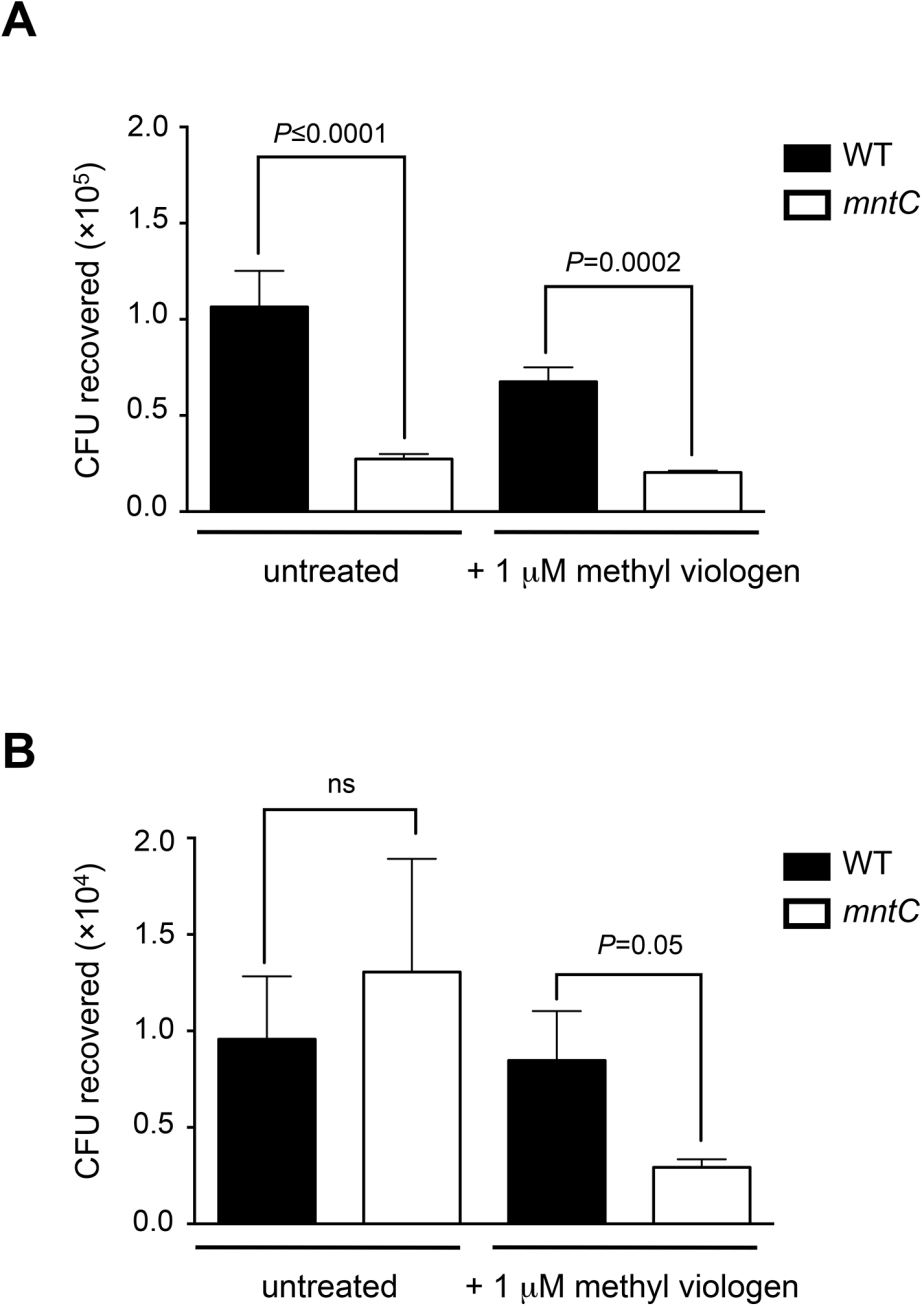
Lack of a functional MntABC system renders *S*. *aureus* more sensitive to killing by methyl viologen and human neutrophils but not murine macrophages, unless *S*. *aureus* is pre-exposed to oxidative stress. (A, B) Survival of wild-type and *mntC* mutant strains within neutrophils harvested from heparin-treated human blood (A) and INF-γ-activated murine macrophages (B). Bacteria were either untreated or pre-exposed to 1 μM methyl viologen for 1 hour. Neutrophils (A) and macrophages (B) were lysed after 90 min and 24 hours of infection, respectively, to enumerate CFU. Bars represent the mean value of triplicate samples and error bars are standard deviation. *P-*values were determined using one-way ANOVA with multiple comparisons between samples via Tukey’s post-test.

Previous studies showed that *S*. *aureus* cells lacking a functional MntABC system are more susceptible to killing by methyl viologen [10], a ROS-generating quaternary ammonium compound [28]. Since the mechanism of ROS generation by methyl viologen has shown to be distinct from the oxidative burst of phagocytic cells [28–31], we wondered whether the higher susceptibility of *S*. *aureus* cells lacking a functional MntABC system to methyl viologen *in vitro* is translated into an increased killing by phagocytic cells during host infection. To this end, we sought to compare the survival of wild-type and *mntC* mutant strains upon exposure to neutrophils and macrophages isolated from A/J mice *ex vivo*. Due to the limited volume of whole blood that can be recovered from mice, we decided to use neutrophils that were isolated from human donors instead of murine neutrophils for our experiment. Human neutrophils were infected with either the opsonized wild-type or *mntC* mutant strain at a multiplicity of infection (MOI) of 10:1 and the number of viable intracellular bacteria was determined 90 min later. Compared to the wild-type strain, the *mntC* mutant strain was statistically significantly more susceptible to killing by neutrophils (Fig 1A). Notably, we observed increased killing of the *mntC* mutant only when neutrophils were isolated from whole blood that was treated with heparin, but not with EDTA, as an anticoagulant (data not shown). Several studies have reported that treatment of whole blood with EDTA results in significantly reduced ROS production by neutrophils compared to heparinized whole blood [35, 36]. It is possible that the increased sensitivity of the *mntC* mutant to the phagocytic oxidative burst manifests only at a high amount of ROS, similar to sensitivity to killing by methyl viologen (S1 Fig). Next, we tested the survival of wild-type and *mntC* mutant strains within macrophages. Interferon-gamma (IFN-γ) activated bone-derived murine macrophages were infected with either wild-type or *mntC* mutant cells at a MOI of 10:1 and the number of viable intracellular bacteria was determined 24 hours later. In contrast to the survival within human neutrophils, there was no difference between wild-type and *mntC* mutant strains in killing by activated murine macrophages (Fig 1B).

Several lines of evidence indicate that *S*. *aureus* is able to survive inside phagocytic cells and contribute to infection [37–39]. Moreover, it has been suggested that neutrophil destruction after phagocytosis of USA300 is in part a form of programmed necrosis [38]. Therefore, it is likely that surviving *S*. *aureus* cells experience repetitive cycles of oxidative burst during infection. We wondered whether *S*. *aureus* cells that were previously phagocytosed and have already experienced an oxidative burst become more susceptible to killing during a second round of phagocytosis. To pre-expose bacteria to oxidative stress before phagocytic infection we incubated bacteria with a sub-lethal concentration of methyl viologen in which the survival rate of both strains was equal (S2 Fig), since it was not feasible to recover a sufficient number of intracellular bacteria to perform a second round of infection. Pre-treatment with a sub-lethal concentration of methyl viologen rendered the *mntC* mutant strain significantly more susceptible to killing by murine macrophages, while it had a minor effect on the susceptibility of the *mntC* mutant strain towards neutrophil killing (Fig 1A and 1B). It is possible that the observed difference in the susceptibility of the *mntC* mutant to killing by neutrophils and macrophages is due to differences in the amount and species of ROS produced by these phagocytic cells (cf. Discussion for details).

### *S*. *aureus* cells lacking MntABC have delayed resumption of growth following internalization by phagocytic cells

**Fig 2:**
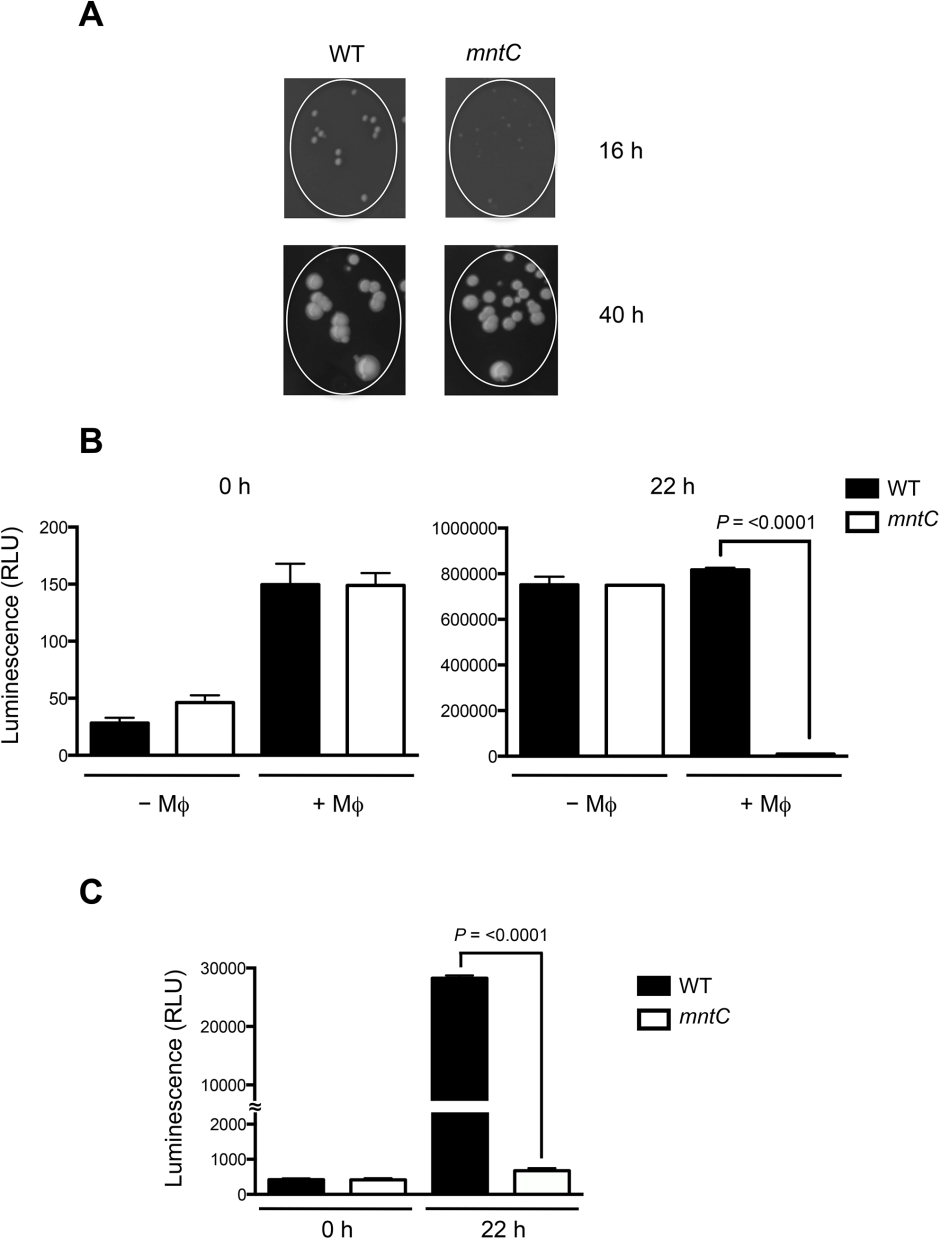
Recovery of the *mntC* mutant is delayed after phagocytosis. (A) Wild-type and *mntC* mutant strains that were harvested from murine macrophages were plated onto agar plates containing 5% defibrinated sheep blood. Images were taken after 16 and 40 hours of incubation at 37°C. (B, C) Growth after phagocytosis by macrophages (B) or neutrophils (C) was measured by BacTiter-Glo (Promega) 22 hours after recovery from phagocytic cells and inoculation into manganese-restricted media. Data are mean values of triplicate samples with standard deviation.

Notably, we observed that the *mntC* mutant strain formed significantly smaller colonies on agar plates compared to the wild-type strain after recovery from phagocytic cells (Fig 2A). After additional incubation at 37°C for 24 h, the colony size reached that of the wild-type strain (Fig 2A), suggesting that the nature of the small *mntC* mutant colonies is distinct from that of small colony variants [40]. Similarly, smaller-sized colonies were observed when the *mntC* strain was recovered from infected kidneys of immune-sufficient mice or cultures containing sub-lethal concentrations of methyl viologen (Figure A in S3 Fig). Since growth in manganese-restricted media alone did not result in smaller *mntC* mutant colonies (Figure B in S3 Fig), we hypothesized that the *mntC* mutant might be impaired in resuming growth after recovery from the oxidative burst of phagocytic cells. To this end, wild-type and *mntC* mutant cells recovered from murine macrophages were inoculated in fresh liquid media and growth was determined by measuring the amount of intracellular ATP, which is indicative of the presence of metabolically active cells [41]. Measurement of the total amount of ATP is significantly more sensitive than the measurement of optical density when analyzing bacterial growth at low cell density [41]. Both the wild-type and *mntC* mutant samples had comparable amounts of total ATP at the time of inoculation into fresh media (Fig 2B). Strikingly, while the number of metabolically active wild-type cells significantly increased after inoculation in liquid culture that of the *mntC* mutant cells recovered from macrophages increased only marginally (Fig 2B). Both wild-type and *mntC* mutant cells proliferated similarly when they were not pre-exposed to murine macrophages (Fig 2B). Similar results were obtained when the growth of wild-type and *mntC* mutant cells recovered from human neutrophils was analyzed (Fig 2C). These results clearly indicate that MntABC is crucial for the growth of *S*. *aureus* cells following phagocytosis.

**Fig 3:**
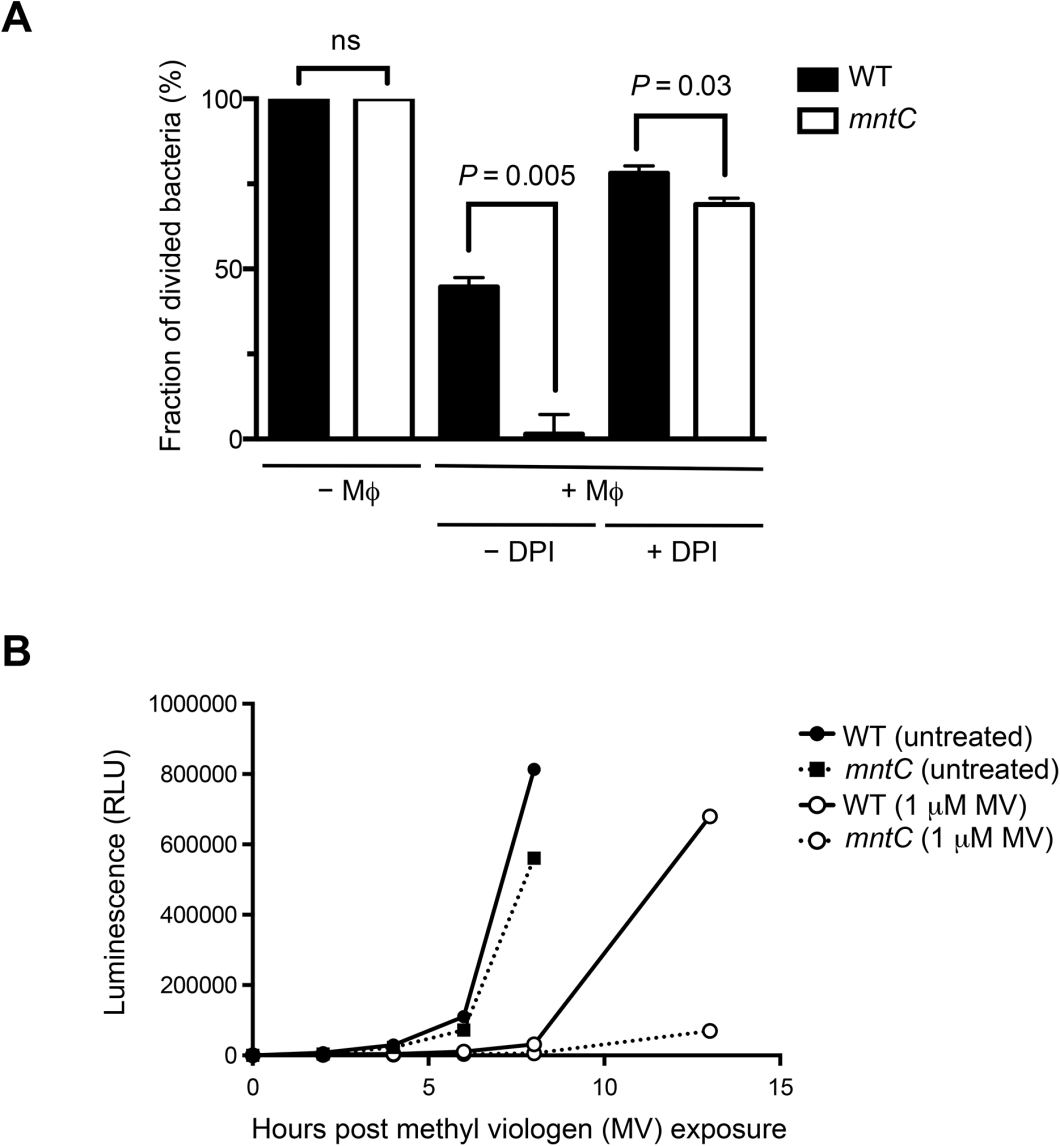
MntABC is important for efficient proliferation of *S*. *aureus* after oxidative stress. (A) Percentage of wild-type and *mntC* mutant cells that lost CFSE fluorescence when inoculated into TSB media after recovery from IFN-γ activated murine macrophages with and without DPI treatment. Bacteria were identified by cell size and further differentiated from macrophage debris by co-staining with an anti-*S*. *aureus* antibody. Data are median values from triplicate samples with standard deviation. (B) Wild-type and *mntC* mutant cells were treated with 1 μM methyl viologen (MV) for 1 hour. Growth after treatment was measured at indicated time points for 12 hours post exposure, via an increase in BacTiter-Glo luminescence values. Once bacterial cultures reached stationary phase growth, growth was no longer measured. Data are mean values of triplicated samples with standard deviation.

To confirm that MntABC is important for reinitiation of *S*. *aureus* replication following internalization by phagocytic cells we ascertained the proportion of wild-type and *mntC* bacteria that have started to divide by labeling the bacteria with the fluorescent dye carboxyfluorescein diacetate succinimidyl ester (CFSE) prior to infection of murine macrophages. CFSE covalently binds to intracellular amines and as cells divide CFSE segregates equally among daughter cells with each division; thus, diminishing signal intensity, as measured by flow cytometry, is indicative of cell division [42]. As a proof of concept, we monitored proliferation of CFSE-labeled wild-type *S*. *aureus* with or without irradiation exposure, which inhibits cell division. While non-irradiated cells started to lose CFSE labeling soon after inoculation into liquid culture *in vitro*, irradiated cells fully retained CFSE labeling for at least 24 hours indicating that the CFSE label is stably maintained in the absence of cell division (Figure A in S4 Fig). In addition, CFSE-labeled wild-type bacteria that were inoculated into a saturated overnight culture of unlabeled wild-type bacteria, which inhibit the growth of CFSE-labeled bacteria, retained the CFSE signal significantly longer compared to cells that were inoculated into a fresh growth media (S5 Fig) Moreover, flow cytometry analysis of CFSE-labeled wild-type and *mntC* mutant bacteria at hourly intervals yielded a series of overlapping, normally distributed curves reflecting uniform replication within the bacterial population and confirming that *mntC* mutant bacteria, which were not previously exposed to phagocytic cells, have a comparable growth rate with that of wild-type bacteria (Fig 3A, Figure B in S4 Fig). In contrast, when *mntC* mutant cells were recovered from macrophages, they had a significant growth defect as was evident by the significant retention of CFSE fluorescence, which is indicative of non-dividing cells (Fig 3A).

### Delay in replication of the *mntC* strain is associated with oxidative stress

To determine whether the delay in replication of the *mntC* mutant strain was a consequence of the oxidative stress experienced inside macrophages, we inhibited the oxidative burst of IFN-γ-activated macrophages with diphenylene iodonium (DPI), which is a well-known inhibitor of NADPH oxidase [43]. The DPI treatment of IFN-γ activated macrophages prior to infection diminished the previously observed growth defect of *mntC* mutant cells (Fig 3A) suggesting that the growth defect of the *mntC* mutant strain was primarily a consequence of the phagocytic oxidative burst.

We used methyl viologen to confirm that the growth defect of *mntC* mutant cells that suffered oxidative stress *ex vivo* can be reproduced *in vitro*. To this end, we monitored the growth of both wild-type and *mntC* mutant strains after exposure to a sub-lethal concentration of methyl viologen at which the survival rates of both strains are equivalent (S2 Fig). Without pre-exposure to methyl viologen, both strains had comparable growth profile (Fig 3B). Pre-treatment with methyl viologen substantially extended the lag-phase of both wild-type and *mntC* mutant strains (Fig 3B). Notably, while the wild-type strain had grown substantially by 13 hours after inoculation into fresh media, the growth of the *mntC* mutant strain was still significantly impaired (Fig 3B). Together, our results demonstrate that a functional MntABC system is crucial for *S*. *aureus* to recommence growth after enduring oxidative stress.

### Genes involved in DNA damage repair are highly upregulated in *S*. *aureus* cells lacking MntABC after phagocytosis

**Fig 4:**
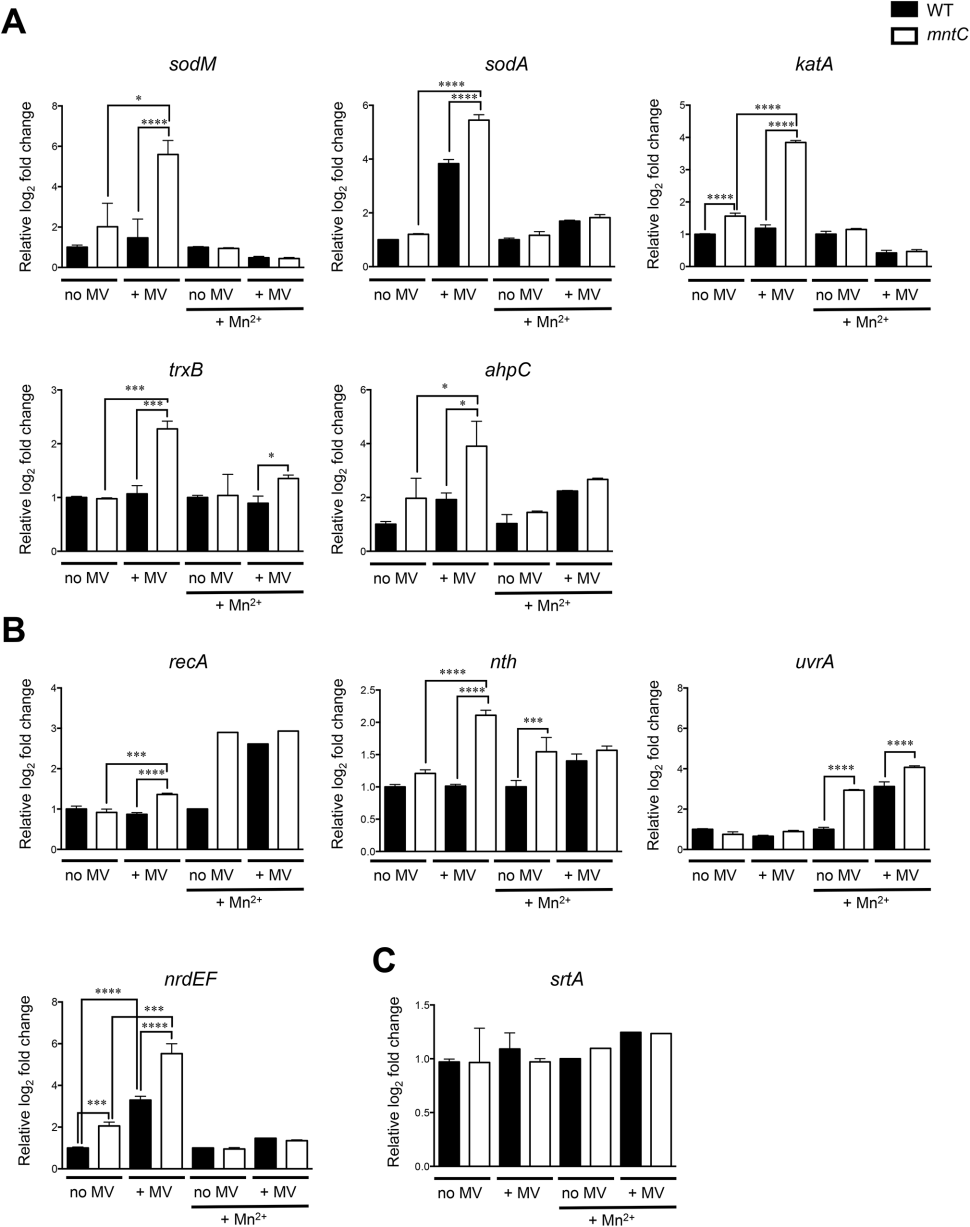
Genes involved in oxidative stress response and DNA repair are highly upregulated in the *mntC* mutant after exposure to a sub-lethal concentration of methyl viologen *in vitro*. Expression of genes involved in oxidative stress response (A), DNA repair (B) and the *srtA* gene (C) was determined via qRT-PCR in wild-type (filled bars) and *mntC* (open bars) cells grown in RPMI-H media and treated with or without 1 μM methyl viologen (MV) for 1 hour. Bars represent the mean value of triplicate samples and error bars are standard deviation. *P*-values (* = <0.05, *** = <0.001, **** = <0.0001) were determined using student’s t-test.

In order to better understand the molecular mechanism underlying the severe growth defect of *S*. *aureus* cells lacking MntABC after the phagocytic oxidative burst, we sought to investigate the differences in the cellular response of the wild-type and *mntC* mutant strains to oxidative stress. Bacterial survival under oxidative stress conditions relies on a series of distinct defense mechanisms including detoxification of ROS through enzymes and repair of damaged macromolecules, in particular DNA, which was shown to be the most sensitive target of oxidative damage [22, 44]. Therefore, we aimed to study the differences in the transcription of genes involved in these two key processes between wild-type and *mntC* mutant strains under oxidative stress conditions (Table 1). First, we performed quantitative real-time reverse transcription PCR (qRT-PCR) analysis on bacteria that were exposed to a sub-lethal concentration of methyl viologen *in vitro*. The methyl viologen-exposed *mntC* mutant bacteria consistently upregulated genes involved in oxidative stress resistance and DNA repair/synthesis compared to those that were not treated with methyl viologen (Fig 4A and 4B). Moreover, the expression levels of genes involved in oxidative stress resistance and DNA repair/synthesis in the *mntC* mutant strain were significantly higher compared to those of the wild-type strain (Fig 4A and 4B). Addition of excess manganese repressed the induction of the majority of the genes involved in oxidative stress resistance and DNA repair/synthesis in the *mntC* mutant strain upon exposure to a sub-lethal concentration of methyl viologen (Fig 4A and 4B). The expression of *srtA*, which encodes for an enzyme involved in anchoring LPXTG-proteins to the cell wall and whose expression is unrelated to oxidative stress [45], was comparable between wild-type and *mntC* mutant strains and between methyl viologen exposed and non-exposed bacteria (Fig 4C).

**Table 1:** List of genes analyzed by qRT-PCR.

Gene	Name	Locus tag	GeneID	Function	**Mn** ^**2+**^ **dependency**	**Essential**	**Reference**
*sodM*	superoxide dismutase	SAUS300_0135	3913957	oxidative stress response	requires Mn^2+^ as a cofactor	no	25, 47
*sodA*	superoxide dismutase	SAUS300_1513	3915273	oxidative stress response	requires Mn^2+^ as a cofactor	no	25, 48
*katA*	catalase A	SAUSA300_1232	3914723	oxidative stress response	regulated through PerR regulon	no	46, 47
*trxB*	thioredoxin-disulfide reductase	SAUSA300_0747	3913567	oxidative stress response	regulated through PerR regulon	no	46
*ahpC*	alkyl hydroperoxide reductase subunit C	SAUSA300_0380	3914843	oxidative stress response	regulated through PerR regulon	no	46, 47
*nrdE*	ribonucleotide diphosphate reductase subunit αlpha	SAUSA300_0716	3913497	DNA repair/synthesis		yes	49
*nrdF*	ribonucleotide diphosphate reductase subunit beta	SAUSA300_0717	3913498	DNA repair/synthesis	requires Mn^2+^ as a cofactor	yes	49
*recA*	recombinase A	SAUSA300_1178	3913385	DNA repair/synthesis		no	52
*nth*	endonuclease III	SAUSA300_1343	3912944	DNA repair/synthesis		no	51
*uvrA*	excinuclease ABC subunit A	SAUSA300_0742	3913512	DNA repair/synthesis		no	51
*srtA*	sortase A	SAUSA300_2467	3913526	cell wall biosynthesis		no	45

Expression of the *katA*, *trxB* and *ahpC* genes is controlled by the peroxide resistance regulon repressor PerR, whose activity depends on intracellular metal concentrations, in particular those of iron and manganese [46, 47]. Since a significant increase in the expression of these three genes in the *mntC* mutant strain was only observed when bacteria were exposed to a sub-lethal concentration of methyl viologen, low intracellular manganese concentration alone likely is not sufficient to induce the expression of genes regulated by the PerR operon (Fig 4A and 4B). Among the genes tested, three genes encode manganese-dependent enzymes: SodM, SodA and NrdF [48, 49, 50]. Their enzymatic activity is expected to be impaired in the *mntC* mutant strain due to a non-functional MntABC system. Despite the fact that all the genes involved in oxidative stress response and DNA repair/synthesis tested were highly upregulated in the *mntC* mutant strain, the *mntC* mutant strain exposed to a sub-lethal concentration of methyl viologen had a significant growth defect when bacteria were inoculated into fresh media lacking methyl viologen (Fig 3B). This suggests that the proper function of manganese-dependent enzymes during oxidative stress is likely crucial for protecting *S*. *aureus* cells from oxidative damage and ensuring efficient growth after oxidative stress.

**Fig 5:**
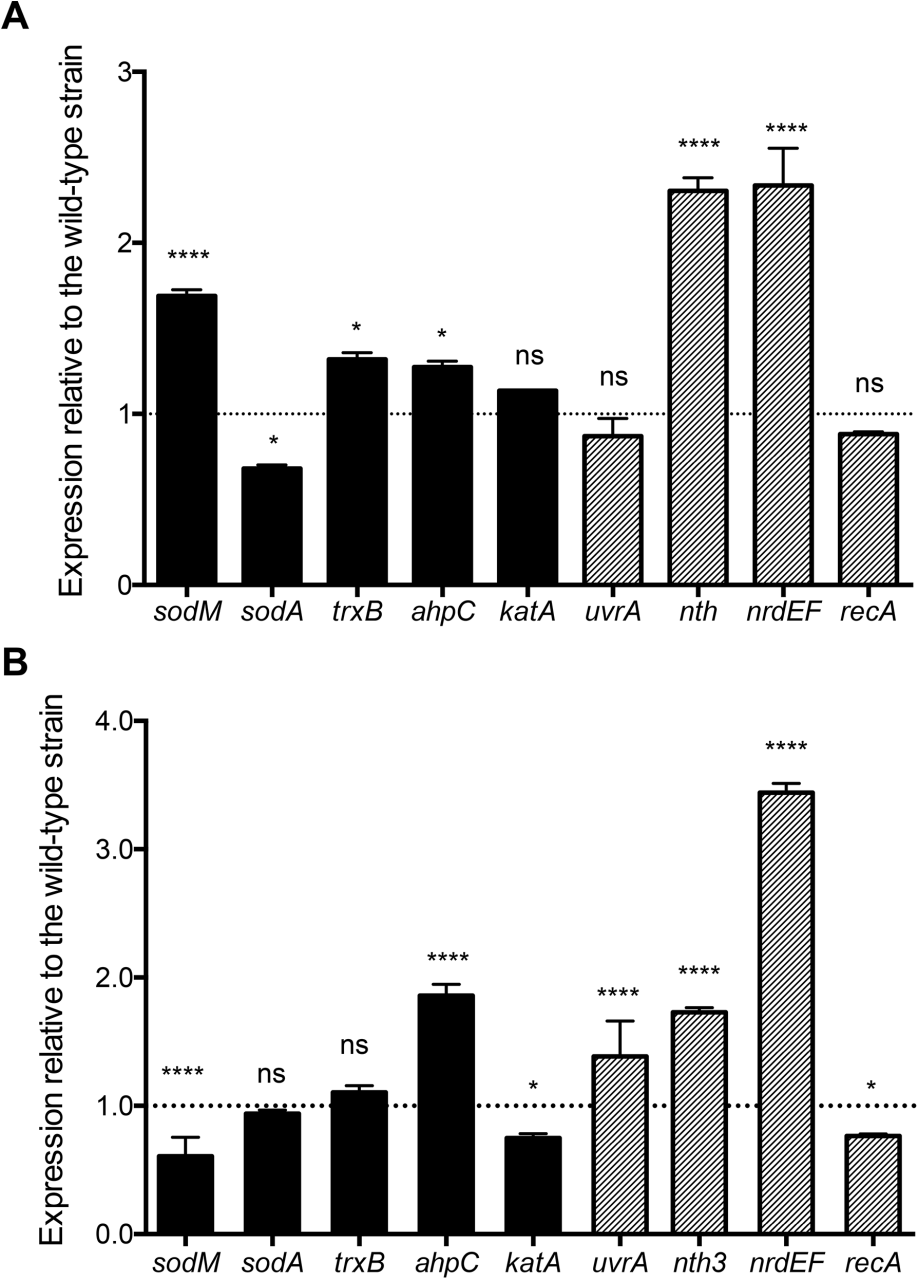
The *nrdEF* genes are highly upregulated in the *mntC* mutant after exposure to murine macrophages and human neutrophils *ex vivo*. Expression of genes involved in oxidative stress response (filled bars) and DNA repair (striped bars) in the *mntC* mutant strain relative to the wild-type strain that were phagocytosed by murine macrophages for 2 hours (A) or human neutrophils for 45 minutes (B). Expression levels were determined via qRT-PCR. Bars represent the mean value of triplicate samples and error bars are standard deviation. *P*-values (ns = non-significant, * = <0.05, *** = <0.001, **** = <0.0001) were determined using student’s t-test.

Next, we analyzed whether similar transcriptional differences between wild-type and *mntC* mutant strains are observed upon phagocytosis *ex vivo*. In contrast to bacteria exposed to a sub-lethal concentration of methyl viologen *in vitro*, *nrdEF* were the only genes that were induced 2-fold more in the *mntC* mutant strain than the wild-type strain after exposure to both murine macrophages and human neutrophils (Fig 5A and 5B). *nth*, which encodes for a DNA repair enzyme [51] was highly induced in murine macrophages, but not in human neutrophils. The gene encoding for the protein RecA [52] was induced neither in murine macrophages nor human neutrophils. The *nrdEF* genes, which are transcribed together [50], encode the NrdEF class Ib ribonucleotide reductase complex that catalyzes the conversion of nucleotides to deoxynucleotides, providing the monomeric building blocks required for DNA replication and repair [53].

**Fig 6:**
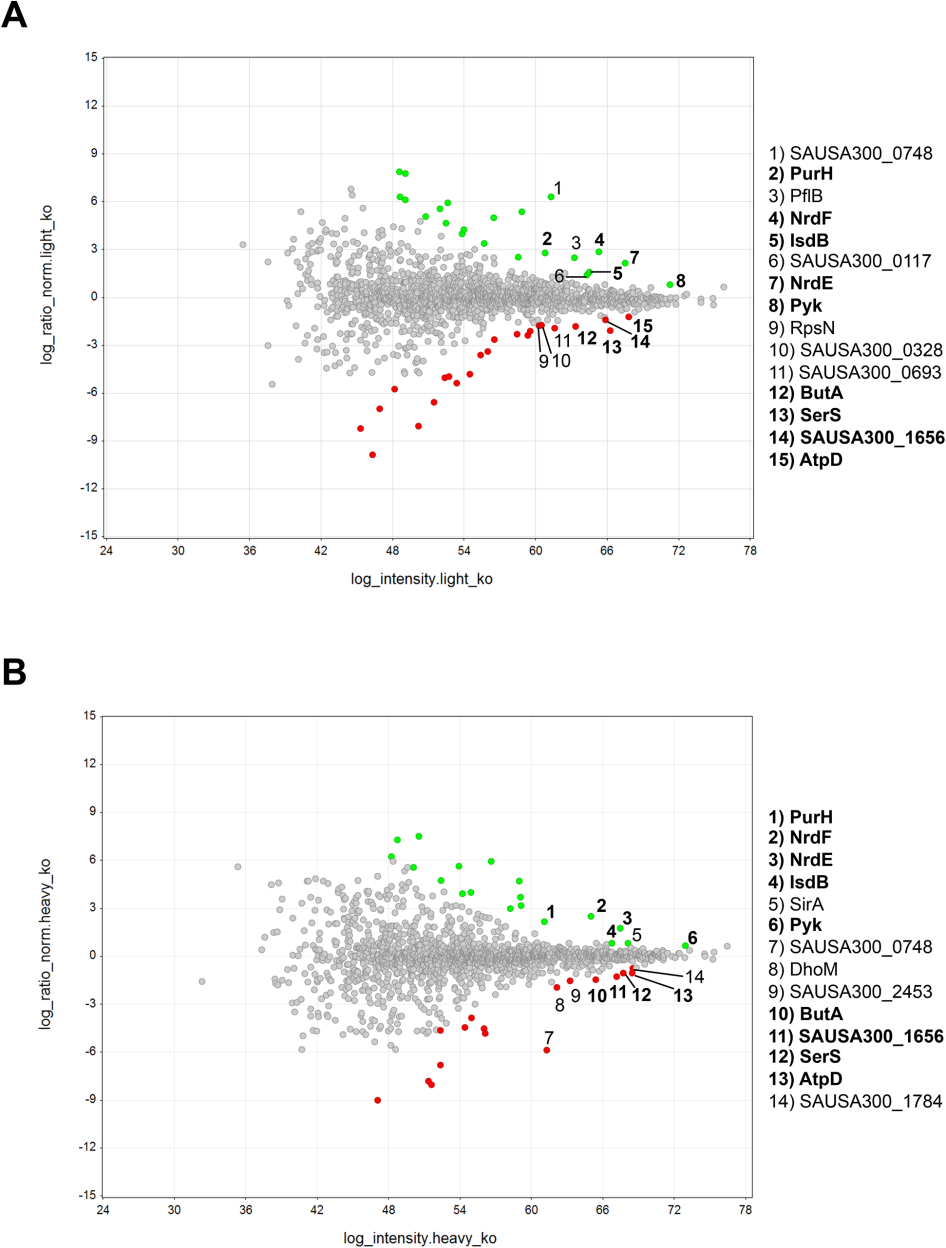
The NrdEF proteins are highly induced in the *mntC* mutant when exposed to oxidative stress *in vitro*. (A) MA plot of the log_2_ transformed normalized ratio of “light” *mntC* mutant relative to the wild-type strain against the log_2_ transformed signal intensity arising from the “light” *mntC* mutant. (B) MA plot of the log_2_ transformed normalized ratio of “heavy” *mntC* mutant relative to the wild-type strain against the log_2_ transformed signal intensity arising from the “heavy” *mntC* mutant. Proteins with z-scores for ratios between the *mntC* mutant and wild-type strains in all replicates greater than 2 and smaller than −2 are shown in green and red dots, respectively. Proteins that were identified both in (A) and (B) that had the strongest mass spectrometric signals are shown in bold.

In order to determine whether the observed upregulation of the *nrdEF* genes directly translates to an increased protein level and if other proteins are specifically induced or repressed in the *mntC* mutant strain compared to the wild-type strain in response to oxidative stress, we took a non-biased proteomics approach to compare the protein abundance between the wild-type and *mntC* mutant strains under oxidative stress conditions using stable isotope labeling by amino acids in cell culture (SILAC) [54]. Proteomic analysis requires a large number of bacterial cells, thus limiting our analysis to bacteria grown *in vitro*. The wild-type and *mntC* strains were grown in the presence of a sub-inhibitory concentration of methyl viologen in liquid culture containing either natural (“light”) amino acids or non-radioactive, stable isotope containing (“heavy”) amino acids. When “light” and “heavy” cell populations are mixed, they remain distinguishable by mass spectrometry (MS) and protein abundances are determined from the relative MS signal intensities by LC-MS/MS analysis [54]. We selected proteins that were identified and quantified with a minimum of 2 distinct peptide observations, with the greatest overall mass spectrometric signal levels which had relative abundance ratios 2 or more standard deviations from the mean (Fig 6A and 6B, S2 and S3 Tables). Of those, we focused on proteins that were most upregulated or downregulated in both biological replicas (Fig 6A and 6B, S2 and S3 Tables). 5 proteins were more abundant in the *mntC* strain compared to the wild-type strain and 4 proteins were present at lower abundance in the *mntC* mutant strain compared to wild-type (Fig 6A and 6B). Strikingly, NrdE and NrdF were among the most highly upregulated proteins in the *mntC* mutant strain (Fig 6A and 6B, S2 and S3 Tables). PurH, which is involved in the biosynthesis of purines, the pyruvate kinase Pyk, and IsdB were also more abundant in the *mntC* strain compared to wild-type (Fig 6A and 6B, S2 and S3 Tables). IsdB is known to be the primary receptor for hemoglobin, but has also been reported to play a role in promoting resistance to hydrogen peroxide and neutrophil killing [55]. Among the proteins that were less abundant in the *mntC* mutant are SerS, which is involved in protein biosynthesis, as well as ButA and AtpD that function in general energy metabolism [32] (Fig 6A and 6B, S2 and S3 Tables). SerS is likely to be essential for *S*. *aureus* viability as no transposon mutants have been identified so far [56–59]. Further studies are needed to determine whether its down regulation has any effect on *S*. *aureus* fitness and whether the growth defect of the *mntC* mutant strain after oxidative stress is in part caused by the down regulation of this essential gene.

Overall, our transcript and proteomic analyses revealed that the manganese-dependent NrdEF protein complex, which is essential for DNA synthesis and repair, might play a critical role in maintaining *S*. *aureus* fitness under oxidative stress conditions. Since NrdEF requires manganese as a cofactor, its function is likely impaired in the *mntC* mutant strain in the absence of a functional uptake manganese uptake system. The observed induction of NrdEF, both on the mRNA and protein level, in the *mntC* mutant strain could be an attempt of bacteria to compensate for the impaired function of NrdEF by increasing the protein copy number. NrdEF proteins were shown to be essential for the viability of *S*. *aureus* [56–59]. Therefore, an impaired protein function during infection would be detrimental to the growth and survival of the pathogen inside the host.

## Discussion

Increasing evidence suggests that the ability of *S*. *aureus*, in particular MRSA, to survive within and eventually escape from phagocytic cells contributes to its dissemination and pathogenesis within the host [37–39]. *S*. *aureus* readily causes lysis of both neutrophils and macrophages through the production of leukocidins, which allows the bacteria to not only survive inside phagocytes, but also to escape [60–62]. In order to thrive in the host, *S*. *aureus* cells that have survived the first oxidative burst of primary phagocytic cells need to rapidly recover from oxidative stress and prevent further oxidative damage by potential secondary phagocytosis events. In this study, we showed that MntABC plays a critical role in ensuring efficient growth of *S*. *aureus* cells after phagocytic oxidative burst.

The role of manganese in detoxifying ROS is well established and it has been anticipated that an important role of manganese acquisition in the pathogenesis of *S*. *aureus* is to protect bacteria from killing by phagocytic cells. Our analysis revealed that the *mntC* mutant strain was indeed highly susceptible to the ROS-generating reagent methyl viologen *in vitro* (S1 Fig); however, the *mntC* mutant was only marginally more susceptible to phagocytic killing *ex vivo* compared to the wild-type strain (Fig 1A and 1B). A possible explanation is that the mechanism of ROS generation by methyl viologen and oxidative burst of phagocytic cells is different. Methyl viologen causes redox cycling, i.e. a repetitive cycle of oxidation and reduction, so that ROS generation is continuous [28, 29]. In contrast, the oxidative burst of phagocytic cells represents a rapid, transient release of ROS [30, 31]. Therefore, the amount of ROS that bacteria experience *in vitro* in the presence of methyl viologen is expected to be significantly higher than *in vivo* and the greater susceptibility of the *mntC* mutant to ROS might become more obvious under conditions where high amounts of ROS accumulates. Interestingly, we showed that the *mntC* mutant strain was killed more rapidly by neutrophils than macrophages. This may be due to differences in the antimicrobial mechanisms of these two phagocytic cell types. For example, neutrophils kill *S*. *aureus* more rapidly than macrophages [63]. This is partly attributed to an increased intensity of oxidative burst in neutrophils compared to macrophages [63–68]. HOCl, which is a strong oxidant, is produced by neutrophils, but not by macrophages due to the lack of myeloperoxidase [18].

Although the presence of a functional MntABC system did not fully protect *S*. *aureus* cells from phagocytic killing (Fig 1A and 1B), it played a significant role in ensuring efficient bacterial growth after phagocytosis (Fig 2A–2C). It seems likely that in the absence of a functional MntABC system, *S*. *aureus* cells endure more oxidative damage due to an impaired ability to acquire a sufficient amount of manganese as evidenced by a profound delay in resuming growth after oxidative stress (Fig 3A and 3B). Although the exact physiological and molecular processes underlying bacterial lag phase are not fully understood, the repair of macromolecular damage [69] and the synthesis of cellular components necessary for growth [70] are thought to be critical. There are two possible mechanisms by which the uptake of manganese could influence the processes underlying the lag phase. The first involves the reduction of ROS-mediated cellular damage, as manganese plays a key role in detoxifying ROS [23, 71]. *S*. *aureus* possesses at least two different manganese-dependent superoxide dismutases, SodM and SodA, which play distinct roles in counteracting oxidative stress depending on bacterial growth phase and the nature of oxidative stress [48, 49]. A previous study has demonstrated that the loss of functional manganese acquisition system in *S*. *aureus* results in reduced superoxide dismutase activity when the availability of manganese is restricted [11]. The second mechanism involves the repair of cellular damage induced by ROS. Our transcript analysis revealed that among the genes involved in ROS detoxification and DNA repair tested, *nrdEF* are the highest upregulated genes in the *mntC* mutant strain relative to the wild-type strain upon phagocytosis (Fig 5A and 5B). Notably, proteomic analysis showed that the NrdEF proteins were the most highly induced proteins in the *mntC* mutant strain relative to the wild-type strain after exposure to a sub-lethal concentration of methyl viologen *in vitro* (Fig 6A and 6B). Since NrdEF requires manganese as a cofactor, its function is likely impaired in the *mntC* mutant strain. The observed induction of NrdEF, both on the mRNA and protein level, in the *mntC* mutant strain could be an attempt by the bacteria to compensate for the impaired function of NrdEF by increasing the protein copy number. NrdEF proteins have been shown to be essential for the viability of *S*. *aureus* [56–59]. Therefore, the potentially impaired function of the protein during infection would be detrimental to the growth and survival of the pathogen inside the host. Additionally, a recent study revealed that the NrdEF complex is important for the replication of *E*. *coli* under oxidative stress conditions when the iron concentration is low as found in the host [72].

Overall, our results suggest that a functional manganese acquisition system is not only important for ROS detoxification, as has been previously proposed, but also for repairing oxidative damage. The severe growth defect of the *mntC* mutant strain after exposure to oxidative stress is therefore likely a consequence of increased damage due to an impaired activity of manganese-dependent superoxide dismutases [11] and the decreased ability in repairing the damage due to an impaired function of the manganese-dependent NrdEF complex.

## Supporting Information

S1 Fig Lack of a functional MntABC system renders *S*. *aureus* more sensitive to killing by high concentrations of methyl viologen. Survival of wild-type and *mntC* mutant strains after overnight incubation with methyl viologen at different concentrations. In addition, survival of the *mntC* strain in which the stop-codon was repaired, and that of the *mntC* strain in the presence of 5 μM MgCl_2_ is shown.
(TIF)

S2 Fig Survival of wild-type and *mntC* mutant strains after treatment with a sub-lethal concentration of methyl viologen. Wild-type and *mntC* mutant strains were incubated in 1 μM methyl viologen in RPMI-H for 1 hour. The initial inoculum as well as the number of viable cells post-treatment was determined by plating samples onto agar plates containing 5% defibrinated sheep’s blood. Relative CFU/mL (survival) was determined by dividing cell count post-treatment by the cell count of the initial inoculum.
(TIF)

S3 Fig The growth of the *mntC* mutant is delayed after exposure to a sub-lethal concentration of methyl viologen, but indistinguishable from that of the wild-type strain when not exposed to oxidative stress. Wild-type and *mntC* mutant strains were exposed to 1 μM methyl viologen for 1 hour (Figure A) or grown in RPMI-H (Mn-low media) (Figure B) and plated onto agar plates containing 5% defibrinated sheep blood. Images were taken after 16 hours of inoculation at 37°C.
(TIF)

S4 Fig Flow cytometry analysis of CFSE-labeled wild-type and *mntC* mutant cells. Histogram plots demonstrating the influence of irradiation on the loss of CFSE fluorescence in wild-type cells grown in TSB media (Figure A). Histogram plots showing the loss of CFSE fluorescence in wild-type or *mntC* mutant cells grown in TSB media (Figure B).
(TIF)

S5 Fig Flow cytometry analysis of CFSE-labeled wild-type bacteria. CFSE-labeled wild-type bacteria were diluted into the fresh TSB media (Figure A) or saturated overnight culture of unlabeled bacteria (Figure B). The cultures were shaken at 37°C and samples were taken at indicated time points for FACS analysis.
(TIF)

S6 Fig Susceptibility of wild-type and *mntC* mutant strains towards H_2_O_2_ and HOCl. Survival of wild-type and *mntC* mutant strains after overnight incubation in 40 μM H_2_O_2_ and 5.5 mg/ml NaOCl. The number of viable bacteria was determined by BacTiter-Glo luminescence measurement (Figure A) or by CFU determination (Figure B). Data are from triplicate samples and error bars are standard deviation. * indicates statistical significance (*P*-values = <0.05) based on student’s t-test.
(TIF)

S1 Table Primers used for qRT-PCR. (PDF)

S2 Table Proteins identified in the reverse labeled lysates with "Heavy" wild-type and "Light"*mntC*. (PDF)

S3 Table Proteins identified in the labeled lysates with "Light" wild-type and "Heavy"*mntC*. (PDF)
