# Supplementary figures and images for: The Staphylococcus aureus ABC-Type Manganese Transporter MntABC Is Critical for Reinitiation of Bacterial Replication Following Exposure to Phagocytic Oxidative Burst
Source: PLoS One. 2015 Sep 17;10(9):e0138350. doi: 10.1371/journal.pone.0138350 (PMC4574778; doi:10.1371/journal.pone.0138350)

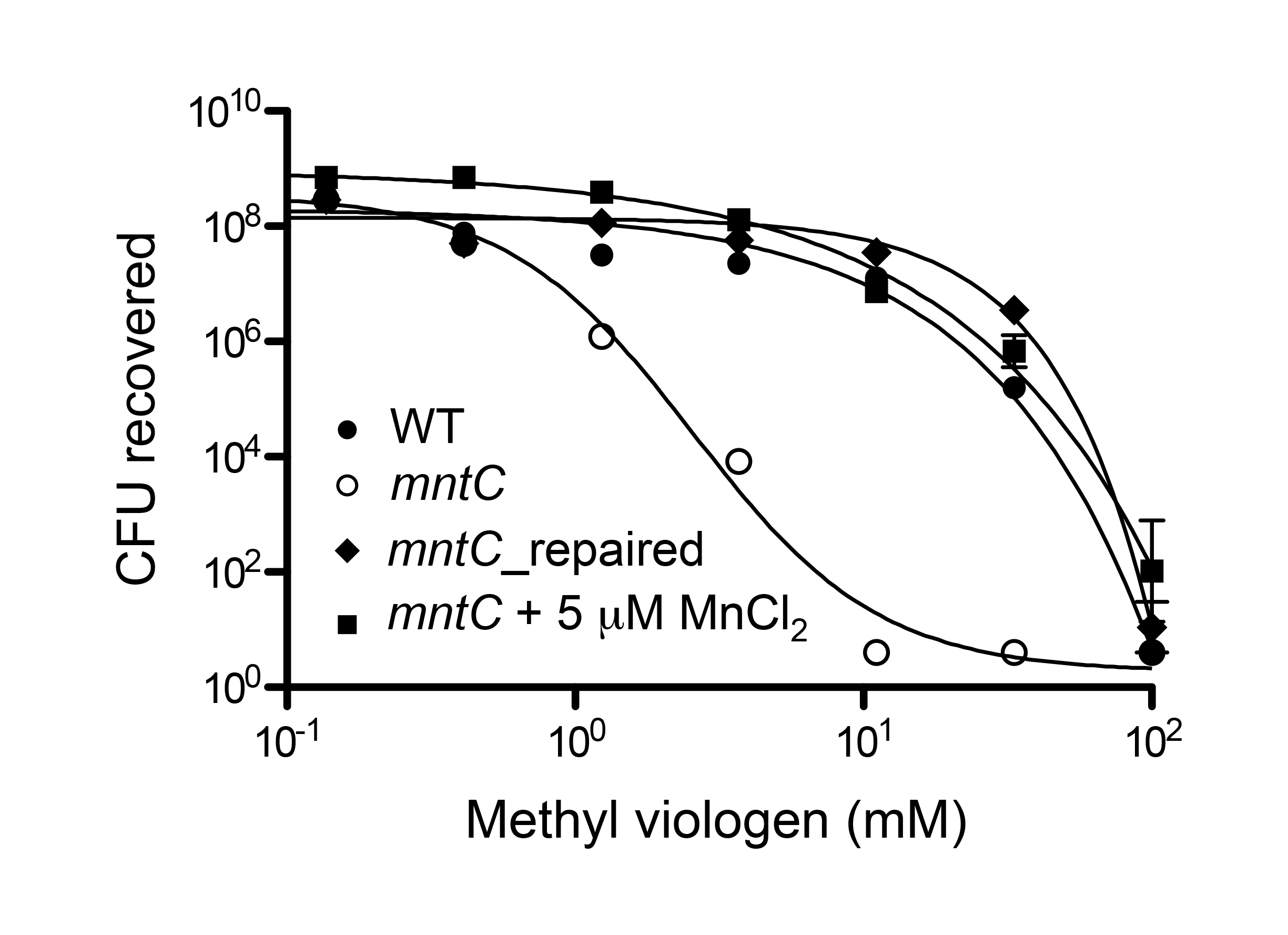

Supplement: S1 Fig — Survival of wild-type and mntC mutant strains after overnight incubation with methyl viologen at different concentrations. In addition, survival of the mntC strain in which the stop-codon was repaired, and that of the mntC strain in the presence of 5 μM MgCl2 is shown. (TIF) [file pone.0138350.s001.tif]

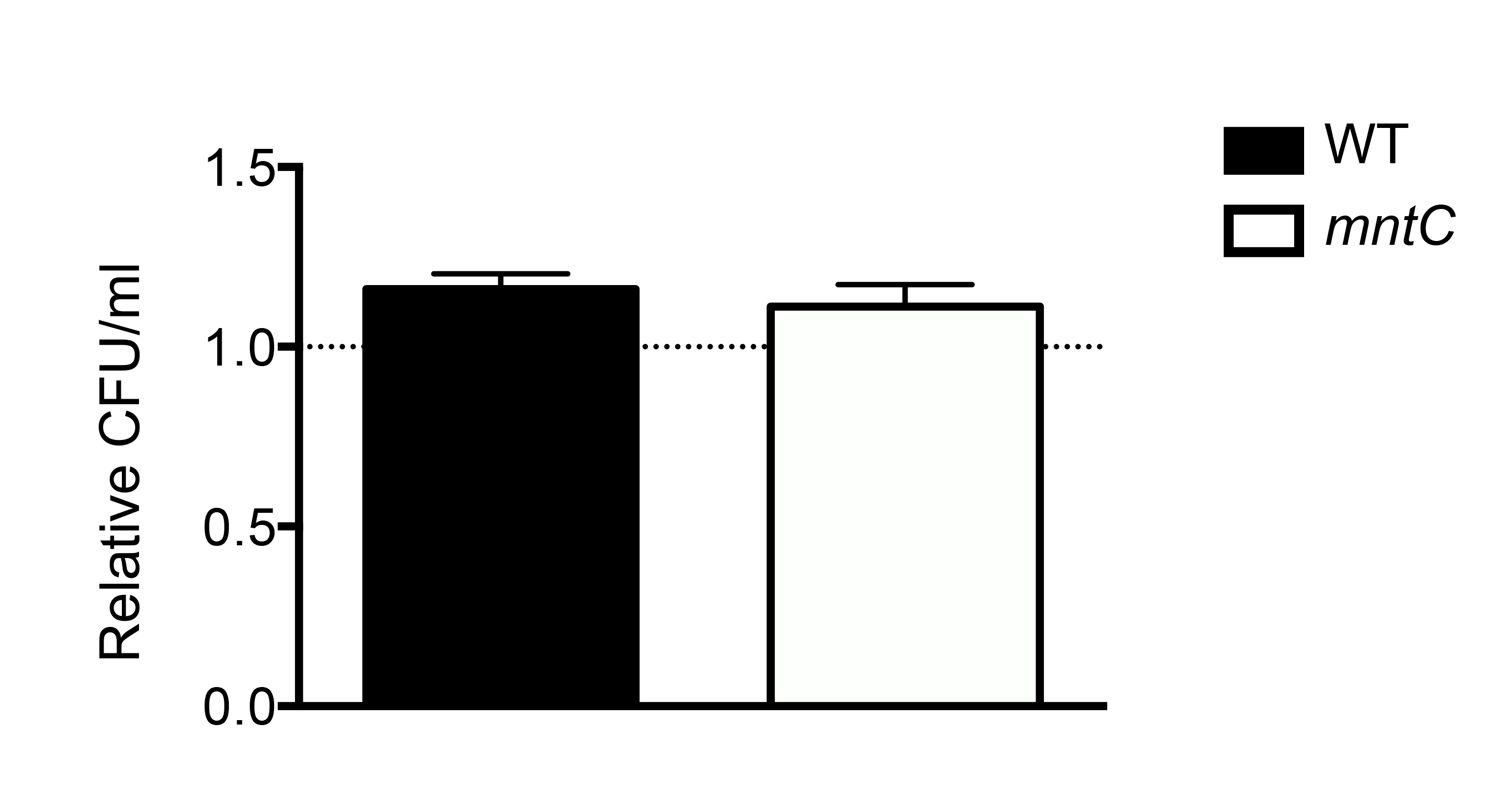

Supplement: S2 Fig — Wild-type and mntC mutant strains were incubated in 1 μM methyl viologen in RPMI-H for 1 hour. The initial inoculum as well as the number of viable cells post-treatment was determined by plating samples onto agar plates containing 5% defibrinated sheep’s blood. Relative CFU/mL (survival) was determined by dividing cell count post-treatment by the cell count of the initial inoculum. (TIF) [file pone.0138350.s002.tif]

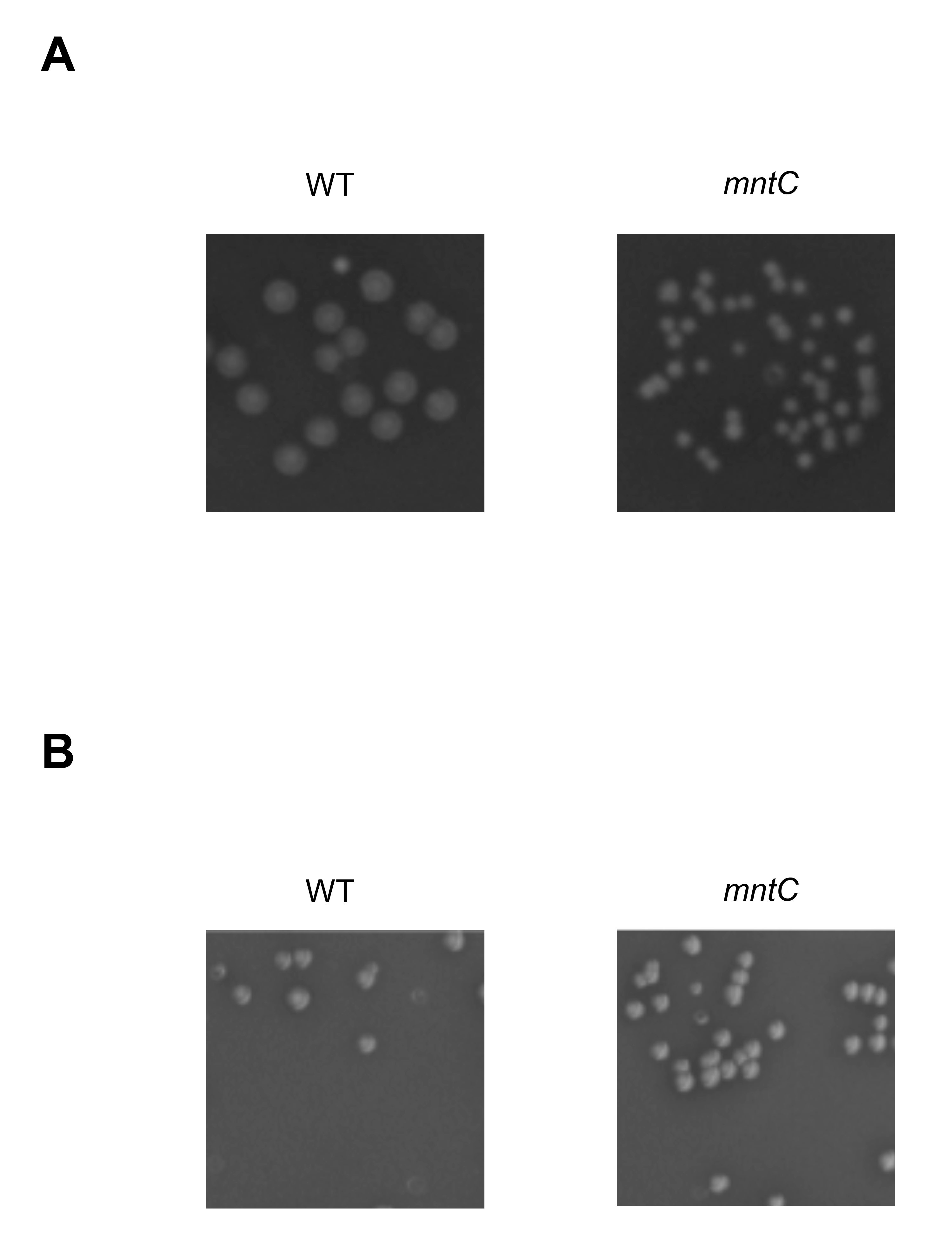

Supplement: S3 Fig — Wild-type and mntC mutant strains were exposed to 1 μM methyl viologen for 1 hour (Figure A) or grown in RPMI-H (Mn-low media) (Figure B) and plated onto agar plates containing 5% defibrinated sheep blood. Images were taken after 16 hours of inoculation at 37°C. (TIF) [file pone.0138350.s003.tif]

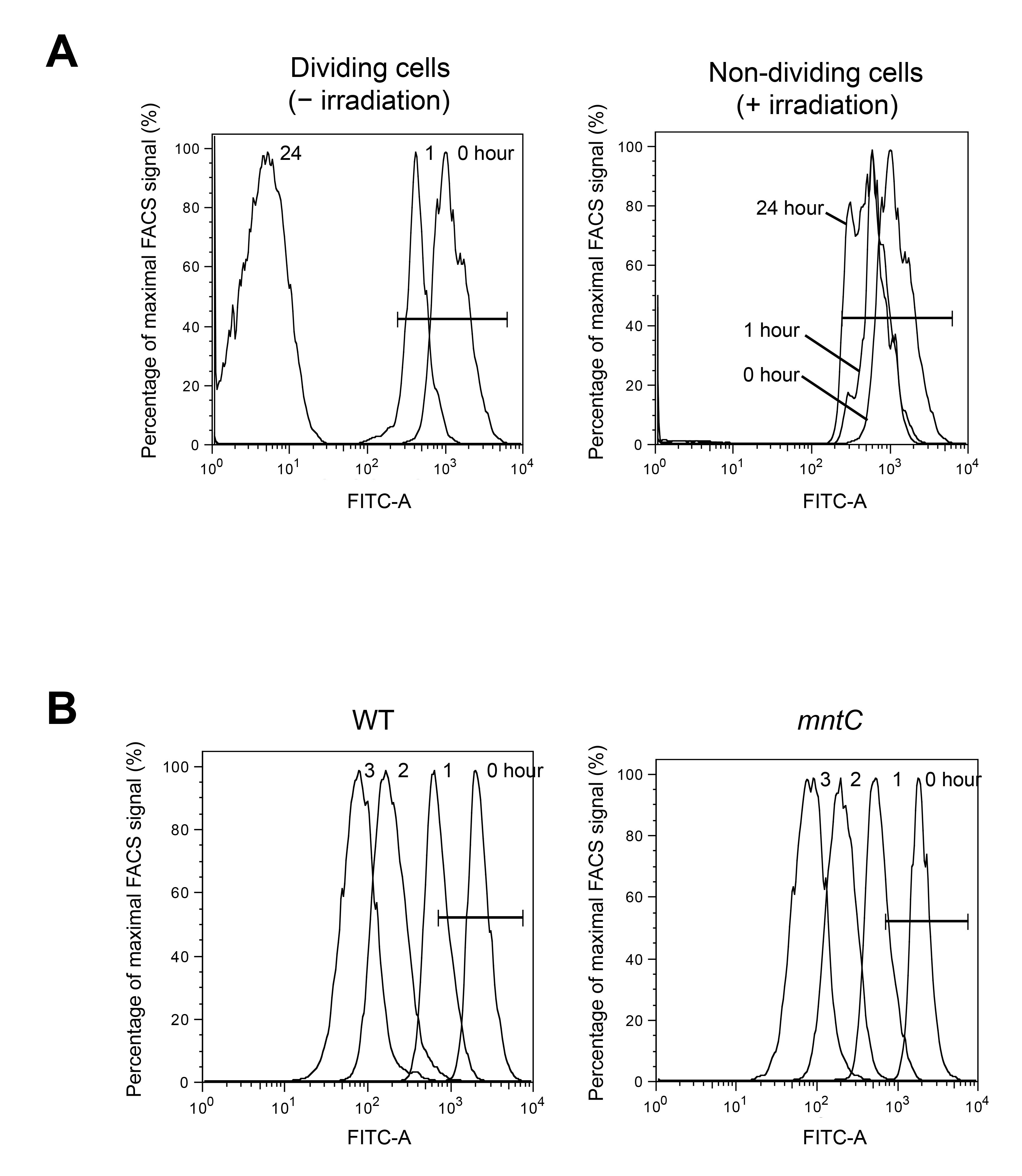

Supplement: S4 Fig — Histogram plots demonstrating the influence of irradiation on the loss of CFSE fluorescence in wild-type cells grown in TSB media (Figure A). Histogram plots showing the loss of CFSE fluorescence in wild-type or mntC mutant cells grown in TSB media (Figure B). (TIF) [file pone.0138350.s004.tif]

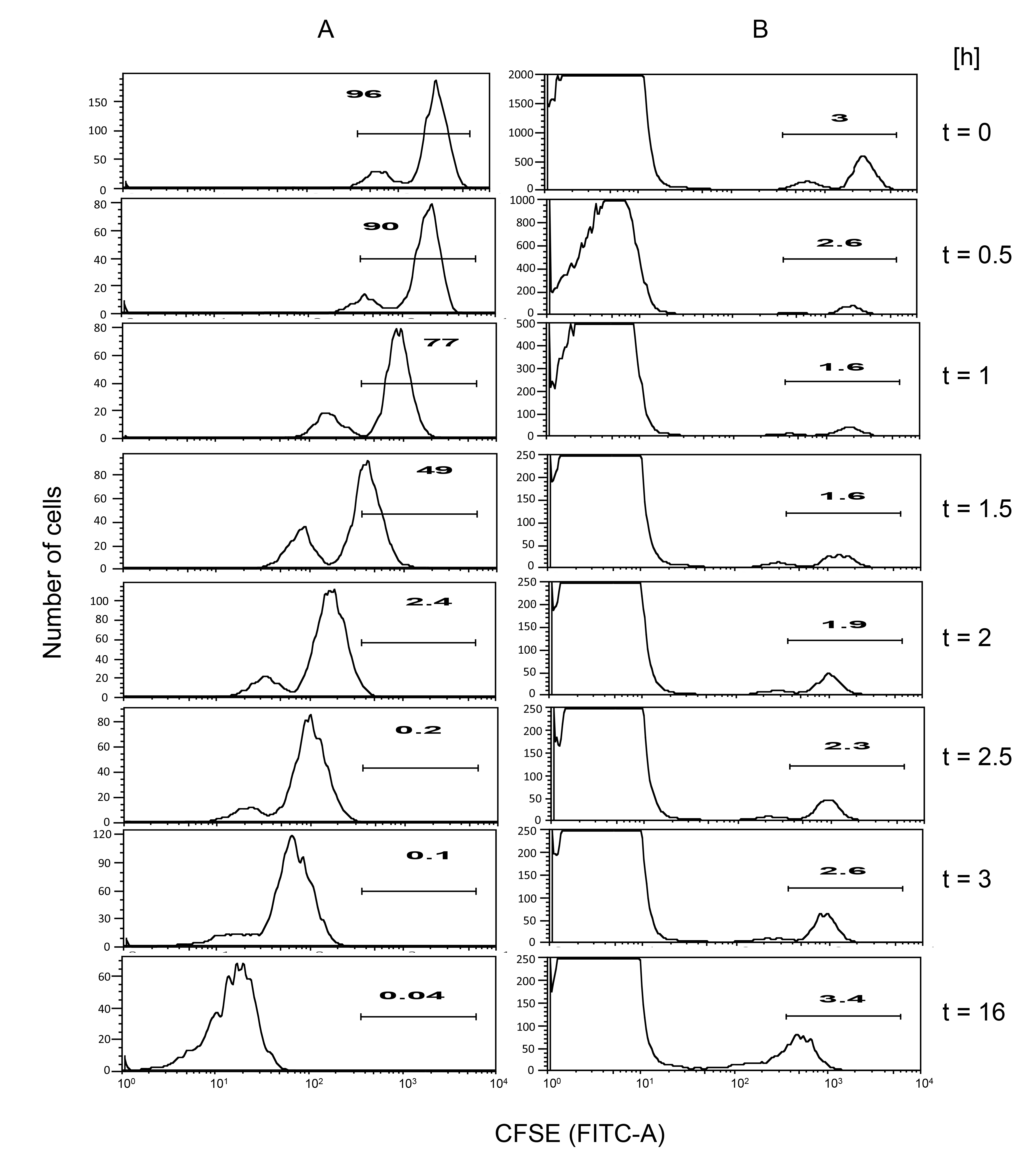

Supplement: S5 Fig — CFSE-labeled wild-type bacteria were diluted into the fresh TSB media (Figure A) or saturated overnight culture of unlabeled bacteria (Figure B). The cultures were shaken at 37°C and samples were taken at indicated time points for FACS analysis. (TIF) [file pone.0138350.s005.tif]

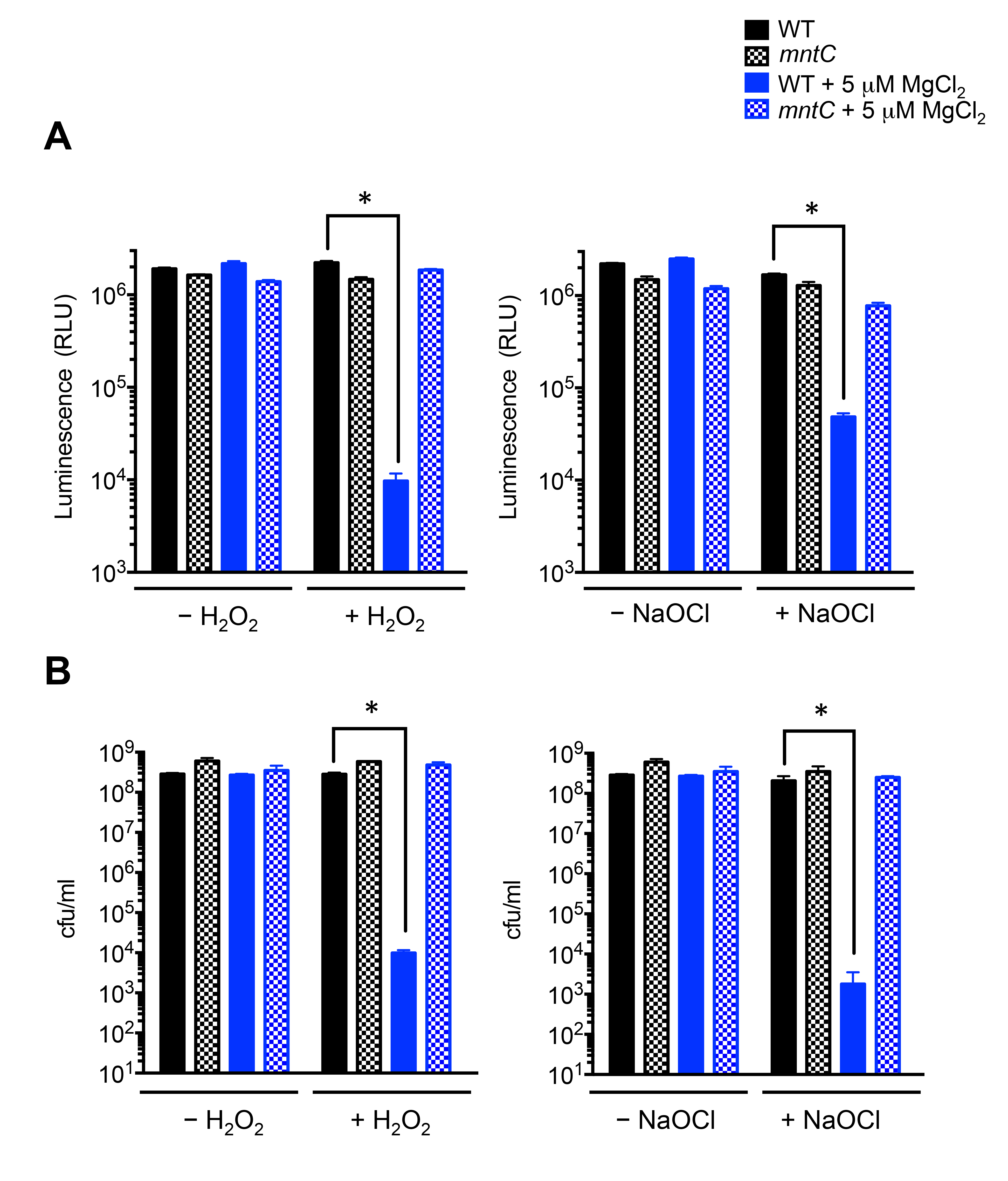

Supplement: S6 Fig — Survival of wild-type and mntC mutant strains after overnight incubation in 40 μM H2O2 and 5.5 mg/ml NaOCl. The number of viable bacteria was determined by BacTiter-Glo luminescence measurement (Figure A) or by CFU determination (Figure B). Data are from triplicate samples and error bars are standard deviation. * indicates statistical significance (P-values = <0.05) based on student’s t-test. (TIF) [file pone.0138350.s006.tif]
