# Supplementary material for: The Staphylococcus aureus ABC-Type Manganese Transporter MntABC Is Critical for Reinitiation of Bacterial Replication Following Exposure to Phagocytic Oxidative Burst
Source: PLoS One. 2015 Sep 17;10(9):e0138350. doi: 10.1371/journal.pone.0138350 (PMC4574778; doi:10.1371/journal.pone.0138350)
Supplement: S1 Table — (PDF) [file pone.0138350.s007.pdf]

Table S1 Primers used for qRT-PCR

Gene	Forward primer sequence	Reverse primer sequence	Reporter (FAM) sequence
recA	CGTTACGTAAACTTTCAGGTGCTATTTCTA	GTGTAGTCTCTGGATTACCGAACAT	TCATCAACCAAATTCG
uvrA	TGGTGAAGCACAACGTATTCGATTA	GCAGTCCAATTGATGGCTCATCTAATA	ACGCGACCCAATTTG
trxB	GCAGTATGTGATGGTGCATTCTTT	GAATGTTCCCTCTTCTACTGCTGAA	ACCACCACCGATAACG
sodM	TGAAGAAAAAGGTGGCGTAATAGATGA	CCAAGTCCAACCTGATCCAAATAATGT	CCACTGCGCTTTGATG
nth	TGGAGAAATACCACAAACACATAAGGAAT	TTCATCAAATGCTACACTCATGACTACA	CACCTGCTAAACTTTC
nrdEF	GGTTGCAAACCCAAATGTTGAGAAA	CCTGATTGTAATTGTGTTTGCGCAAT	TTTCACGCGCATTCTT
katA	GCGTTTGCACCAACTAATATTATTCCA	GCGCATCGCCATATGAGAATAAAC	CCTTGCAGCATTTTGT
sodA	AGTGTACCAGCTAACATCCAAACTG	GTGAAAGTAACTCCCAGAATAATGAATGG	CCACCGCCATTATTAC
ahpC	AGACGGTGTTGTACAAGCATCTG	GTACTAGCGTCACGGCCAATT	CCGTCAGCGTTAATTT
srtA	GCAAGCTAAACCTCAAATTCCGAAA	GCTGGTCCTGGATATACTGGTTCTTTAATAT	TAGCCTGCCACTTTCG
rrsA	GGTCTGTAACTGACGCTGATGTG	GTGGACTACCAGGGTATCTAATCCT	TCCCCACGCTTTCG
