# Supplementary material for: The Staphylococcus aureus ABC-Type Manganese Transporter MntABC Is Critical for Reinitiation of Bacterial Replication Following Exposure to Phagocytic Oxidative Burst
Source: PLoS One. 2015 Sep 17;10(9):e0138350. doi: 10.1371/journal.pone.0138350 (PMC4574778; doi:10.1371/journal.pone.0138350)
Supplement: S2 Table — (PDF) [file pone.0138350.s008.pdf]

Table S2 Proteins identified in the reverse labeled lysates with "Heavy" wild-type and "Light"*mntC*

Protein symbol	Gene names	Observations	Log2 ratio (Δ*mntC*/WT)	*z*-score	Log2 summed signal intensity
C5N6C1_STAA3	pyk	1311	0.82	2.50	71.26
C5N3I0_STAA3	nrdE	731	2.14	4.36	67.52
C5N4L2_STAA3	isdB	602	1.61	2.37	64.49
C5N2N2_STAA3	atpD	414	-1.20	-2.17	67.81
C5N193_STAA3	pflB	343	2.49	3.01	63.23
C5N0T1_STAA3	serS	272	-2.07	-3.75	66.28
C5N4F5_STAA3	purH	262	2.78	3.12	60.81
C5N018_STAA3	SAUSA300_2412	182	1.41	2.08	64.31
C5N3I1_STAA3	nrdF	159	2.88	4.36	65.28
C5N6D3_STAA3	SAUSA300_1656	136	-1.39	-2.08	65.86
C5N019_STAA3	SAUSA300_2413	86	4.99	3.72	56.51
C5N584_STAA3	SAUSA300_1226	80	-2.39	-2.57	59.33
C5N592_STAA3	rpsN	55	-1.77	-2.03	60.30
C5N3F4_STAA3	SAUSA300_0693	52	-1.94	-2.32	61.61
C5N101_STAA3	SAUSA300_0129	47	-1.82	-2.16	63.36
C5N0Z8_STAA3	SAUSA300_0126	41	3.38	2.35	55.67
C5N1L3_STAA3	SAUSA300_0328	36	-1.72	-2.01	60.49
C5N3L7_STAA3	SAUSA300_0748	36	6.32	7.53	61.29
C5N6S7_STAA3	SAUSA300_1797	36	5.37	5.38	58.85
C5N425_STAA3	SAUSA300_0848	34	3.99	2.36	53.81
C5N1B0_STAA3	ldh	33	2.52	2.33	58.53
C5N585_STAA3	thrC	33	-2.30	-2.20	58.46
C5N3S1_STAA3	SAUSA300_0798	32	-3.61	-2.63	55.40
C5N403_STAA3	SAUSA300_0828	17	-3.38	-2.48	55.99
C5N694_STAA3	hemC	15	-2.63	-2.05	56.55
C5MZ66_STAA3	SAUSA300_2161	14	-4.82	-2.97	54.48
C5N1G2_STAA3	essC	13	4.27	2.62	53.96
C5N6B7_STAA3	SAUSA300_1639	9	5.95	2.96	52.61
C5MYM6_STAA3	SAUSA300_1875	9	5.09	2.14	50.76
C5N3Y2_STAA3	SAUSA300_1388	8	-5.03	-2.45	52.37
C5N6F3_STAA3	SAUSA300_1677	8	6.13	2.27	49.09
C5N132_STAA3	SAUSA300_0158	7	4.68	2.30	52.48
C5N0T0_STAA3	hutH	5	-8.06	-3.15	50.17
C5N467_STAA3	SAUSA300_0888	5	-4.97	-2.50	52.71
C5MZ94_STAA3	SAUSA300_2148	5	-6.58	-2.93	51.45
C5MZC1_STAA3	secY	4	5.55	2.57	51.99
C5MZ45_STAA3	ilvD	3	-6.98	-2.27	46.91
C5MYR1_STAA3	pcrB	3	-8.22	-2.50	45.31
C5N493_STAA3	SAUSA300_0915	3	7.90	2.86	48.57
C5MYT1_STAA3	SAUSA300_1908	3	6.30	2.29	48.63
C5N2L9_STAA3	SAUSA300_2070	3	-5.36	-2.98	53.37
C5N0A0_STAA3	feoB	2	-9.88	-3.09	46.33
C5N258_STAA3	SAUSA300_2359	2	7.77	2.87	49.06
