# Supplementary material for: The Staphylococcus aureus ABC-Type Manganese Transporter MntABC Is Critical for Reinitiation of Bacterial Replication Following Exposure to Phagocytic Oxidative Burst
Source: PLoS One. 2015 Sep 17;10(9):e0138350. doi: 10.1371/journal.pone.0138350 (PMC4574778; doi:10.1371/journal.pone.0138350)
Supplement: S3 Table — (PDF) [file pone.0138350.s009.pdf]

Table S3 Proteins identified in the labeled lysates with "Light" wild-type and "Heavy"*mntC*

Protein symbol	Gene name	Observations	Log2 ratio (Δ*mntC*/WT)	*z*-score	Log2 summed signal intensity
C5N4Z9_STAA3	tsf	816	0.68	2.23	72.93
C5N4L2_STAA3	isdB	732	0.83	2.05	66.78
C5N3I0_STAA3	nrdE	632	1.76	4.51	67.48
C5N0T1_STAA3	serS	522	-1.04	-2.68	67.72
C5N2N2_STAA3	atpD	484	-1.04	-2.76	68.50
C5N6D3_STAA3	SAUSA300_1656	293	-1.28	-3.35	67.19
C5N4F5_STAA3	purH	203	2.16	2.56	61.11
C5N584_STAA3	SAUSA300_1226	139	-1.94	-2.61	62.14
C5N6R4_STAA3	SAUSA300_1784	122	-0.77	-2.06	68.51
C5N3I1_STAA3	nrdF	115	2.51	4.12	64.99
C5N101_STAA3	SAUSA300_0129	111	-1.48	-2.33	65.40
C5N062_STAA3	SAUSA300_2453	101	-1.52	-2.12	63.26
C5N592_STAA3	rpsN	76	3.19	2.98	59.12
C5N0Y9_STAA3	SAUSA300_0117	75	0.80	2.03	68.13
C5N4X8_STAA3	ffh	56	3.69	3.44	59.07
C5N3L7_STAA3	SAUSA300_0748	39	-5.89	-6.99	61.27
C5N3P6_STAA3	nuc	30	-3.85	-2.20	54.99
C5N0U6_STAA3	SAUSA300_0025	29	-4.82	-3.12	56.13
C5N6S7_STAA3	SAUSA300_1797	28	4.69	4.35	58.97
C5N403_STAA3	SAUSA300_0828	20	2.99	2.43	58.26
C5N2L9_STAA3	SAUSA300_2070	14	5.95	4.10	56.64
C5N345_STAA3	SAUSA300_0589	11	3.98	2.30	54.93
C5N6S8_STAA3	SAUSA300_1798	11	3.92	2.03	54.21
C5MZX3_STAA3	SAUSA300_2370	11	5.65	2.84	53.91
C5MYS3_STAA3	SAUSA300_1898	10	-4.53	-2.92	56.02
C5MYZ3_STAA3	SAUSA300_1960	7	6.25	2.04	48.24
C5N6B7_STAA3	SAUSA300_1639	6	-4.45	-2.35	54.44
C5N6N7_STAA3	SAUSA300_1754	5	-8.05	-3.26	51.62
C5N2Z0_STAA3	SAUSA300_0512	4	7.50	2.90	50.56
C5N309_STAA3	SAUSA300_0560	4	5.56	2.07	50.12
C5N4Q7_STAA3	SAUSA300_1063	4	-6.82	-2.98	52.35
C5N2P5_STAA3	thiE	4	-4.65	-2.03	52.37
C5N148_STAA3	SAUSA300_0174	3	-9.03	-2.90	47.10
C5N331_STAA3	SAUSA300_0579	3	4.72	2.07	52.42
A8Z5P8_STAA3	SAUSA300_0936	3	7.28	2.48	48.76
C5MZX8_STAA3	SAUSA300_2374	3	-7.81	-3.12	51.34
C5MZ69_STAA3	alsS	2	6.36	2.07	47.95
C5N1B9_STAA3	SAUSA300_0243	2	7.69	2.28	45.48
C5N068_STAA3	SAUSA300_2458	2	5.45	2.25	51.88
